# Hematopoietic cell transplantation and cellular therapies in Europe 2021. The second year of the SARS-CoV-2 pandemic. A Report from the EBMT Activity Survey

**DOI:** 10.1038/s41409-023-01943-3

**Published:** 2023-03-06

**Authors:** Jakob R. Passweg, Helen Baldomero, Fabio Ciceri, Selim Corbacioglu, Rafael de la Cámara, Harry Dolstra, Bertram Glass, Raffaella Greco, Donal P. McLornan, Bénédicte Neven, Régis Peffault de Latour, Zinaida Perić, Annalisa Ruggeri, John A. Snowden, Anna Sureda

**Affiliations:** 1 EBMT Activity Survey Office, Hematology Division, University Hospital, Basel, Switzerland; 2 Unit of Hematology and Bone Marrow Transplantation, IRCCS San Raffaele Scientific Institute, Vita-Salute San Raffaele University, Milan, Italy; 3 Department of Pediatric Hematology, Oncology and Stem Cell Transplantation, University of Regensburg, Regensburg, Germany; 4 Hematology Department, Hospital Universitario de la Princesa, Madrid, Spain; 5 Laboratory of Hematology, Department of Laboratory Medicine, Radboud University Medical Center, Nijmegen, The Netherlands; 6 Klinik für Hämatologie und Stammzelltransplantation, HELIOS Klinikum Berlin-Buch, Berlin, Germany; 7 Department of Haematology, University College London Hospitals NHS Foundation Trust, London, UK; 8 Pediatric Immune-hematology Unit, Necker Children Hospital, Assistance Publique Hôpitaux de Paris, Paris, France; 9 BMT Unit, Department of Hematology, Hospital St. Louis, Paris, France; 10 School of Medicine, University of Zagreb, University Hospital Center Zagreb, Zagreb, Croatia; 11 Department of Haematology, Sheffield Teaching Hospitals NHS Foundation Trust, Sheffield, UK; 12 Clinical Hematology Department, Institut Català d’Oncologia-Hospitalet, Institut d’Investigació Biomèdica de Bellvitge (IDIBELL), University of Barcelona, Barcelona, Spain

**Keywords:** Haematological cancer, Leukaemia

## Abstract

In 2021, 47,412 HCT (19,806 (42%) allogeneic and 27,606 (58%) autologous) in 43,109 patients were reported by 694 European centers. 3494 patients received advanced cellular therapies, 2524 of which were CAR-T treatments, an additional 3245 received DLI. Changes compared to the previous year were CAR-T treatment (+35%), allogeneic HCT +5.4%, autologous HCT +3.9%, more pronounced in non-malignant disorders. Main indications for allogeneic HCT were myeloid malignancies 10,745 (58%), lymphoid malignancies 5127 (28%) and non-malignant disorders 2501 (13%). Main indications for autologous HCT were lymphoid malignancies 22,129 (90%) and solid tumors 1635 (7%). In allogeneic HCT, use of haploidentical donors decreased by −0.9% while use of unrelated and sibling donors increased by +4.3% and +9%. Cord blood HCT decreased by −5.8%. Pediatric HCT increased overall by +5.6% (+6.9% allogeneic and +1.6% autologous). Increase in the use of CAR-T was mainly restricted to high-income countries. The drop in HCT activity reported in 2020 partially recovered in 2021, the second year of the SARS-CoV-2 pandemic. The transplant community confronted with the pandemic challenge, continued in providing patients access to treatment. This annual EBMT report reflects current activities useful for health care resource planning.

## Introduction

The European Society for Blood and Marrow Transplantation (EBMT) published a survey in 1990 [1] describing activity in hematopoietic stem cell transplant (HCT) centers in Europe, updated annually thereafter. The survey spanning 32 years includes patients receiving more than 850,000 transplants. The survey was designed in the form of a single page spreadsheet for ease of reporting and has remained in this format ever since. Many additional features have been added, such as refined disease classification, donor type and stem cell source, information on conditioning intensity and pediatric activity.

HCT is an established procedure for many acquired or inherited disorders of the hematopoietic system, benign or neoplastic, including those of the immune system, and as enzyme replacement in metabolic disorders [2–4]. The activity survey of the EBMT, describing the status of HCT, has become an instrument to observe trends and monitor changes in HCT technology in Europe and neighboring countries [5–15]. The survey, using a standardized structure, captures the numbers of HCT from highly committed participating centers, stratified by indication, donor type and stem cell source over time [16, 17]. https://www.ema.europa.eu/en/documents/scientific-guideline/qualification-opinion-cellular-therapy-module-european-society-blood-marrow-transplantation-ebmt_en.pdf]. In more recent years, the survey also included information on cellular therapies qualifying as medicinal products with hematopoietic cells for uses other than to replace the hematopoietic system [18–27]. The analysis of the survey data since 1990 has illustrated a continued and constant increase in the annual numbers of HCT and transplant rates for both allogeneic and autologous HCT. A drop-in activity was reported in 2020 for the first time, likely driven by the SARS-CoV-2 pandemic [14]. This 2021 survey data show a partial recovery of transplant numbers in the second year of the pandemic. Nonetheless, the appearance of the SARS-CoV-2 pandemic led to a 2–3 year setback in transplant activity.

## Patients and methods

### Data collection and validation

We invited participating centers to report their data for 2021 using the activity survey as shown in Table 1. Patients receiving their first transplant in the survey year are reported by disease, donor type and stem cell source. Additional information on the numbers of subsequent transplants performed due to relapse, rejection, or those that are part of a planned sequential protocol are reported in summative form. Information on the number of patients receiving un-manipulated donor lymphocyte infusions (DLIs), non-myeloablative or reduced intensity HCT, and the number of pediatric HCT were also collected.

**Table 1:** Numbers of HCT in Europe 2021 by indication, donor type and stem cell source.

	TRANSPLANT ACTIVITY 2021																	
	No. of patients																	
	Allogeneic												Autologous			Total		
	Family									Unrelated						Allo	Auto	Total
	HLA-id			Twin	Haplo ≥ 2MM		Other family						BM	BM +				
	BM	PBPC	Cord	all	BM	PBPC	BM	PBPC	Cord	BM	PBPC	Cord	only	PBPC	Cord			
**Myeloid malignancies**	**224**	**2424**	**3**	**3**	**301**	**1593**	**2**	**71**	**1**	**302**	**5692**	**129**	**1**	**226**	**0**	**10,745**	**227**	**10,972**
Acute myeloid leukemia	165	1700	2	1	217	1114	2	43	1	182	3590	106	1	211	0	7123	212	7335
1st complete remission	109	1158	1	1	119	595	2	30	1	121	2070	59	1	182	0	4266	183	4449
not 1st complete remission	39	373	1	0	73	338	0	9	0	32	883	27	0	26	0	1775	26	1801
AML therapy-related or myelodysplasia-related changes	17	169	0	0	25	181	0	4	0	29	637	20	0	3	0	1082	3	1085
Chronic myeloid leukemia	8	93	0	1	4	54	0	5	0	13	217	3	0	4	0	398	4	402
Chronic phase	4	41	0	0	1	24	0	5	0	7	116	2	0	3	0	200	3	203
Not chronic phase	4	52	0	1	3	30	0	0	0	6	101	1	0	1	0	198	1	199
MDS or MD/MPN overlap	44	461	1	1	60	326	0	21	0	98	1385	16	0	9	0	2413	9	2422
MPN	7	170	0	0	20	99	0	2	0	9	500	4	0	2	0	811	2	813
**Lymphoid malignancies**	**251**	**1315**	**2**	**4**	**198**	**890**	**6**	**48**	**0**	**247**	**2113**	**53**	**18**	**22,111**	**0**	**5127**	**22,129**	**27,256**
Acute lymphatic leukemia	226	783	2	1	137	519	3	29	0	210	1188	46	0	56	0	3144	56	3200
1st complete remission	126	565	0	0	51	273	2	21	0	110	825	18	0	53	0	1991	53	2044
not 1st complete remission	100	218	2	1	86	246	1	8	0	100	363	28	0	3	0	1153	3	1156
Chronic lymphocytic leukemia	1	51	0	0	3	28	0	1	0	3	102	0	0	55	0	189	55	244
Plasma cell disorders—MM	3	77	0	2	4	33	1	1	0	2	110	0	6	12,931	0	233	12,937	13,170
Plasma cell disorders—other	0	12	0	0	0	4	0	0	0	1	19	0	0	438	0	36	438	474
Hodgkin lymphoma	4	115	0	0	27	100	1	1	0	4	156	2	7	2288	0	410	2295	2705
Non-Hodgkin lymphoma	17	277	0	1	27	206	1	16	0	27	538	5	5	6343	0	1115	6348	7463
**Solid tumors**	**1**	**11**	**0**	**1**	**6**	**19**	**0**	**0**	**0**	**2**	**12**	**0**	**31**	**1604**	**0**	**52**	**1635**	**1687**
Neuroblastoma	1	2	0	0	5	15	0	0	0	2	1	0	18	525	0	26	543	569
Soft tissue sarcoma/Ewing	0	3	0	0	1	2	0	0	0	0	2	0	0	255	0	8	255	263
Germinal tumors	0	1	0	1	0	0	0	0	0	0	0	0	5	431	0	2	436	438
Other solid tumors	0	5	0	0	0	2	0	0	0	0	9	0	8	393	0	16	401	417
**Non-malignant disorders**	**681**	**344**	**24**	**11**	**140**	**199**	**43**	**45**	**1**	**436**	**500**	**77**	**1**	**502**	**0**	**2501**	**503**	**3004**
Bone marrow failure—SAA	186	131	0	4	36	37	4	4	1	166	141	10	0	1	0	720	1	721
Bone marrow failure—other	62	35	4	0	11	17	5	8	0	50	70	4	0	1	0	266	1	267
Thalassemia	137	33	10	0	6	10	6	1	0	33	48	5	0	16	0	289	16	305
Sickle cell disease	131	95	9	0	32	18	8	4	0	15	11	1	0	5	0	324	5	329
Primary Immune deficiencies	144	31	1	4	45	105	15	18	0	128	153	29	1	7	0	673	8	681
Inherited disorders of Metabolism	19	17	0	3	10	11	5	10	0	40	66	28	0	4	0	209	4	213
Autoimmune disease—MS	0	0	0	0	0	0	0	0	0	0	0	0	0	388	0	0	388	388
Autoimmune disease—SSC	0	0	0	0	0	0	0	0	0	0	0	0	0	52	0	0	52	52
Autoimmune disease—other	2	2	0	0	0	1	0	0	0	4	11	0	0	28	0	20	28	48
Others	34	22	1	2	9	18	3	4	0	14	54	3	0	26	0	164	26	190
**Total patients**	**1191**	**4116**	**30**	**21**	**654**	**2719**	**54**	**168**	**2**	**1001**	**8371**	**262**	**51**	**24,469**	**0**	**18,589**	**24,520**	**43,109**
Re/additional transplants	31	140	2	1	60	352	2	10	0	51	539	29	10	3076	0	1217	3086	4303
**Total transplants**	**1222**	**4256**	**32**	**22**	**714**	**3071**	**56**	**178**	**2**	**1052**	**8910**	**291**	**61**	**27,545**	**0**	**19,806**	**27,606**	**47,412**

In addition, in Table 2, centers reported information on different types of cellular therapies qualifying as advanced therapy medicinal products (ATMP). These therapies result from substantial manipulations of collected cells, whether manufactured by industry centrally or locally by an academic institution.

**Table 2:** Numbers of patients treated with a non HCT cellular therapy in Europe 2021 by indication, donor type and cell source.

Number of patients	DLI	CART		MSC		NK cells		selected/expanded T cells or CIK		Regulatory T cells (TREGS)		Genetically modified T cells		Dendritic cells		Expanded CD34+ cells		Genetically modified CD34+ cells		Other		Total excluding patients receiving DLI treatments	
2021	Allo	Allo	Auto	Allo	Auto	Allo	Auto	Allo	Auto	Allo	Auto	Allo	Auto	Allo	Auto	Allo	Auto	Allo	Auto	Allo	Auto	Allo	Auto
GvHD				280	1					7	1	1		1		1				1	6	291	8
Graft enhancement				25				8	17	2						7		2		152	37	196	54
Autoimmune disease			4	17	8												1					17	13
Genetic disease																			29			0	29
Infection				13		9		135	15	5		7	3							5	3	174	21
Malignancy—ALL		42	337	5		9	1	10		1					5			2		4		73	343
Malignancy—HL/NHL		4	1847				1	5					1	1		1					5	11	1854
Malignancy—Other		12	278			13	3	21	13			2	15		17			3		16	17	67	343
DLI for graft enhancement/failure	848																						
DLI for residual disease	540																						
DLI for relapse	1377																						
DLI per protocol	480																						
**Total**	**3245**	**58**	**2466**	**340**	**9**	**31**	**5**	**179**	**45**	**15**	**1**	**10**	**19**	**2**	**22**	**9**	**1**	**7**	**29**	**178**	**68**	**829**	**2665**

Quality control measures included several independent systems: confirmation of validity of data entered by the center, selective comparison of the survey data with MED-A datasets in the EBMT Registry database and crosschecking with National Registries.

### Participating centers

Since 1990, a directory of HCT centers consisting of both members of the EBMT and non-members, in both European and collaborating non-European countries has been accrued. The directory is updated annually according to the centers current activity. In 2021, 724 centers from 53 countries were contacted (44 European and 9 collaborating countries); of which 694 centers responded. This corresponded to a 96% return rate and included 16% EBMT non-members. Thirty active centers failed to report in 2021. Participating centers are listed in the Supplementary Online Appendix in alphabetical order, by country, city, and EBMT center code, with their reported numbers of first and total HCT, and of first allogeneic and autologous HCT. The WHO regional office definitions were used to classify countries as European or non-European. Nine collaborating non-European countries participated in the 2021 survey: Algeria, Iran, Iraq, Jordan, Lebanon, Nigeria, Saudi Arabia, South Africa, and Tunisia. Their data, 2414 HCT in 2320 patients, from 19 actively transplanting centers made up 5% of the total data set and were included in all analyses.

### Patient and transplant numbers

Wherever appropriate, patient numbers corresponding to the number of patients receiving a first transplant in 2021, and transplant numbers reflecting the total number of transplants performed were listed. The term sibling donor included HLA identical siblings and twins but not siblings with HLA mismatches. Unrelated donor transplants included HCT from matched or mismatched unrelated donors with peripheral blood and bone marrow as a stem cell source but not cord blood HCT. Haploidentical transplants were described as any family member with a full haplotype mismatch. Other family member donors were those related donors that are mismatched to a lesser degree than a full haplotype. For the purpose of the analysis, we added the small number of “other family donor” to haploidentical donor HCT. Additional non-first transplants included multiple transplants defined as subsequent transplants within a planned double or triple autologous or allogeneic transplant protocol, and re-transplants (autologous or allogeneic) defined as unplanned HCT for either rejection, poor-graft function or relapse after a previous HCT.

### Hematopoietic advanced cellular therapies other than hematopoietic cell transplantation

Centers were requested to report patients receiving cellular therapies other than HCT. Hematopoietic advanced cellular therapies were defined as infusion of cells undergoing substantial manipulation after collection, either selection and/or expansion, or genetic modification and thus qualify as investigational or approved advanced therapy medicinal products (ATMPs) according to Regulation (EC) N° 1394/2007. In this context, “substantial” should be understood as referring to the definition included in the Regulation and subsequent regulatory documents and may not reflect the workload assumed by cell processing facilities working in conjunction with clinical programs. Depending on their nature and indications, hematopoietic cellular therapies may be designed to replace or to complement HCT. Administration of non-substantially manipulated hematopoietic cells, such as transplantation of CD34+ selected hematopoietic stem cells were counted as HCT and not as cellular therapy [18]. Similarly, un-manipulated lymphocyte infusions post-HCT were counted as donor lymphocyte infusions (DLI) and not as cellular therapy. Hematopoietic cellular therapies include immune effector cells as defined in FACT-JACIE standards for Hematopoietic Cellular Therapy: “A cell that has differentiated into a form capable of modulating or effecting a specific immune response.” This definition covers CAR-T cells and forms the basis for accreditation requirements in recent EBMT-JACIE recommendations [17, 19].

Hematopoietic cellular therapies were categorized as chimeric antigen receptor T cells (CAR -T); in vitro selected/and or expanded T cells or cytokine activated, such as virus specific T cells; cytokine-induced killer cells (CIK); regulatory T cells (TREGS); genetically modified T cells other than CAR-T; natural killer cells (NK); dendritic cells; mesenchymal stromal cells; in vitro expanded CD34+ cells; and genetically modified CD34+ cells. This survey did not include cells from sources other than hematopoietic tissue. On the other hand, gene therapy protocols, such as those used to treat thalassemia or SCID were included, however numbers have remained low.

### Transplant and cellular therapy rates

Transplant rates, defined as the total number of HCT per 10 million inhabitants were computed for each country, without adjusting for patients receiving their HCT in a foreign country. Cellular therapy rates were defined as the numbers of patients receiving a cellular therapy treatment per 10 million population. Population numbers for the European countries in 2021 were obtained from Eurostats: (https://ec.europa.eu/eurostat) and the World Bank database for the non-European countries: (https://databank.worldbank.org).

### Analysis

Wherever appropriate, the absolute numbers of transplanted patients, number of transplants or transplant rates are shown for specific countries, indications, or transplant techniques. Myeloid malignancy include acute myeloid leukemia (AML), myelodysplastic or myelodysplastic/myeloproliferative neoplasia (MDS or MDS/MPN overlap), myeloproliferative neoplasm (MPN), and chronic myeloid leukemia (CML). Lymphoid malignancy include acute lymphocytic leukemia (ALL), chronic lymphocytic leukemia (CLL), Hodgkin lymphoma (HL), non-Hodgkin lymphoma (NHL) and plasma cell disorders (PCD) (including multiple myeloma (MM) and others). Non-malignant disorders include bone marrow failure (BMF: severe aplastic anemia (SAA) and other BMF), thalassemia and sickle cell disease (HG), primary immune deficiencies (PID), inherited diseases of metabolism (IDM), and autoimmune diseases (AID). Others include histiocytosis and other rare disorders.

## Results

### Participating centers in 2021

Of the 694 centers, 455 (66%) performed both allogeneic and autologous transplants; 223 (32%) restricted their activity to autologous HCT, and 12 (2%) to allogeneic transplants only. Four of the 694 responding centers reported no activity due to renovation or changes within the transplant unit. Within the 690 actively transplanting centers in 2021, 121 (18%) performed transplants on both adult and pediatric patients. An additional 125 (18%) were dedicated pediatric transplant centers and 445 (64%) performed transplants on adults only. Thirty centers failed to report in 2021, which, when compared with previously reported data, accounted for approximately 1000 non-reported HCTs.

### Numbers of patients, transplants, and trends in 2021

**Fig. 1:**
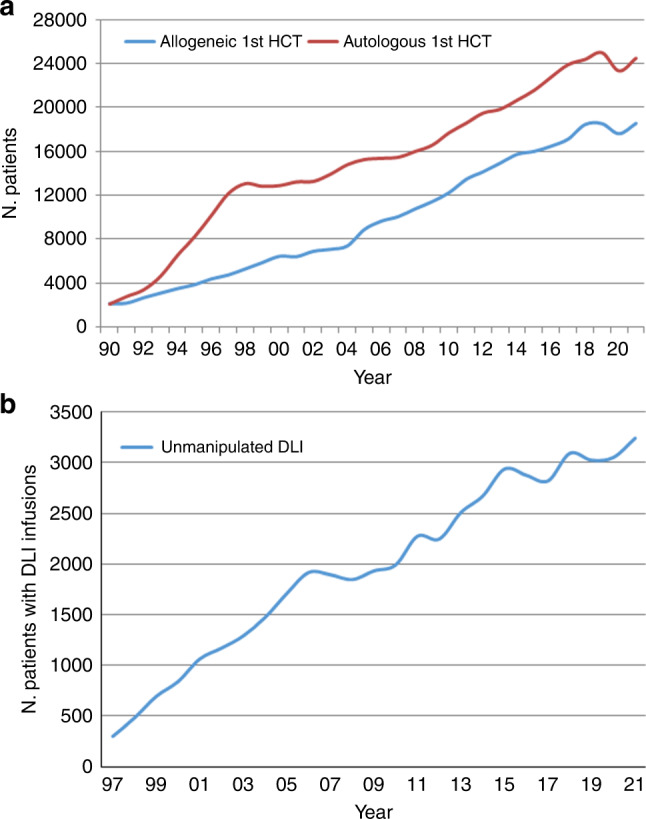
Absolute numbers of patients treated in the years in Europe 1990 to 2021. **a** Absolute numbers of patients receiving their first allogeneic or autologous HCT in Europe 1990–2021. **b** Absolute numbers of patients receiving donor lymphocyte infusions in Europe 1997–2021.

In 2021, 47,412 transplants were reported in 43,109 patients; of these, 19,806 HCT (42%) were allogeneic and 27,606 (58%) autologous (Table 1 and Fig. 1a). After the decrease in HCT activity due to the SARS-CoV-2 pandemic reported in the 2020 survey, the total number of transplants increased again by +4.5% (+5.4% allogeneic HCT and +3.9% autologous HCT) (14). The corresponding number of patients showed an increase of +5.3% for allogeneic HCT and +4.9% for autologous HCT. The use of DLI did not show the same variability in the first year of the SARS-CoV-2 pandemic (Fig. 1b).

In addition, there were 4303 second or subsequent transplants, 1217 being allogeneic, mainly to treat relapse or graft failure and 3086 autologous, the majority of which were part of multiple transplant procedures such as tandem procedures, to treat relapse, or as salvage autologous transplants for PCD. Furthermore, 728 of the allogeneic HCTs were reported as being given after a previous autologous HCT and were mainly for lymphoma or PCD (*n* = 591).

### Pediatric transplantation

The number of pediatric patients (<18 years old at transplant) transplanted in both dedicated pediatric and joint adult-pediatric units was 5437 (4028 allogeneic and 1409 autologous). This is an overall increase of +5.5% in the total number of transplants, with an increase of +6.9% in allogeneic HCT and +1.6% in autologous HCT compared to 2020. Of these, 3832 patients, (2920 allogeneic (76%) and 912 autologous (24%)) were treated in 125 dedicated pediatric centers in 26 countries. Due to the design of the survey, detailed analysis by diagnosis is limited to the dedicated pediatric centers only. Main indications for allogeneic HCT were AML (*n* = 405; 67% in early stage), ALL (*n* = 802; 47% in early stage) and non-malignant disorders (NMD) (*n* = 1352; 37% PID). There were 1588 family and 1332 unrelated donor HCTs reported. Within family donors, 42% were from a haploidentical relative. Bone marrow was used as the stem cell source in 1402 patients of which 62% were family donors. Peripheral blood stem cells were used in 1378 patients with similar proportions seen in both family (*n* = 699) and unrelated donors (*n* = 679). Cord blood stem cells were used in 140 pediatric patients of which 118 (84%) were from unrelated cord blood donors. The main indications for autologous HCT, were solid tumors, with 768 HCT reported in 2021, primarily for neuroblastoma (*N* = 350, 46%).

### Main indications

**Fig. 2:**
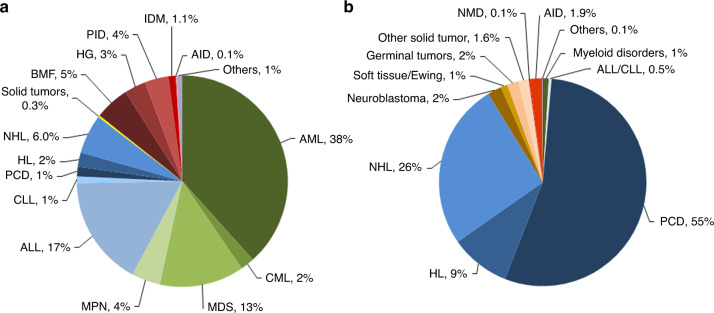
Relative proportion of disease indications for HCT in Europe 2021. Green shades: myeloid malignancies, blue: lymphoid malignancies, brown: solid tumors and red: non-malignant disorders. **a** Allogeneic HCT. **b** Autologous HCT.

Indications for HCT in 2021 are listed in detail in Table 1. Figure 2a, b show the distribution of disease indications for allogeneic (Fig. 2a) and autologous (Fig. 2b) HCT). Main indications for allogeneic HCT were myeloid malignancies; 10, 745 (AML, CML, MDS, MPN, MDS/MPN overlap). For autologous HCT, the main indications were lymphoid malignancies; 22,129 (NHL, PCD, HL, ALL and CLL).

### Changes in allogeneic HCT 2020 to 2021

**Fig. 3:**
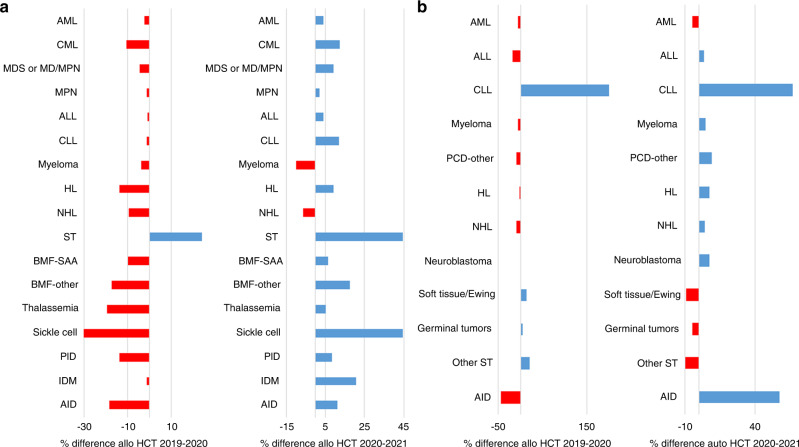
Difference in the percentage of numbers of allogeneic and autologous HCT by diagnosis reported between the years 2019 and 2020 (left) and between the years 2020 and 2021 (right). **a** Allogeneic HCT. **b** Autologous HCT.

In last year’s report on the 2020 transplant activity during the first year of the SARS-CoV-2 pandemic, decreases were seen in the majority of disease indications when compared to 2019. However, this year’s report based on the 2021 activity and despite the ongoing pandemic in many regions in and around Europe, transplant activity increased again in the majority of indications where decreases were previously reported. Figure 3a shows the percentage difference in HCT activity reported by indication between the years 2019 and 2020 (left) and between the years 2020 and 2021 (right). The overall increase in all allogeneic HCT being +5.4% compared to −5.1% in 2020. The leading indication for allogeneic HCT was AML, accounting for 38% of all allogeneic HCT, the decrease of −2.1% reported in 2020 has now increased by +3.9%. Increases were seen in both early stage disease (+6.3%) and therapy-related AML or those with myelodysplasia-related changes (+6.5%). Advanced disease stage however, continued to decrease (−2.9%). Among the myeloid malignancies, CML, which decreased overall by −10.4% in 2020, has increased again by +12.4% to similar numbers seen in 2019. Allogeneic HCT for the chronic myeloid disorders increased by +9.2% for MDS (previously −4.3%) and myeloproliferative neoplasms by +1.9% (previously −1.2%). ALL, comprising 17% of allogeneic HCT, increased overall by +4% when compared to 2020, primarily in early stage (+7.7%) compared to −0.9% in the previous year. CLL increased by +11.8% after a decrease of −1.2% seen in 2020 and HL by +9.3% compared to the −13.6% decrease seen previously. NHL however, continued to decrease by −6.2% (−9.2% in 2020 and −4.1% in 2019) indicating a potential trend towards other treatments. Within the non-malignant disorders, an overall increase (+13%) was seen within all disease indications when compared to the overall decrease of −15% in 2020. BMF - SAA increased by +6.5% (−9.7% in 2020) and BMF non-SAA by +17.7% (−17.2% in 2020). Sickle cell disease increased dramatically by +44.6% (−30.9% in 2020) and thalassemia by +5.1% (−19.6% in 2020). PID increased by +8.4% (−13.6% in 2020) and IDM by +20.8% (−1.1% in 2020). Allogeneic HCT for autoimmune diseases remained a rare indication with just 20 patients treated in 2021. Within allogeneic HCT, 8,071 (41%) were performed using non-myeloablative or reduced intensity conditioning in 2021. Of note, the remarkable decrease of −11% in transplants using myeloablative conditioning seen during 2020 has now increased by +8.3% to similar numbers reported in earlier years.

### Changes in donor type and stem cell source 2020 to 2021

**Fig. 4:**
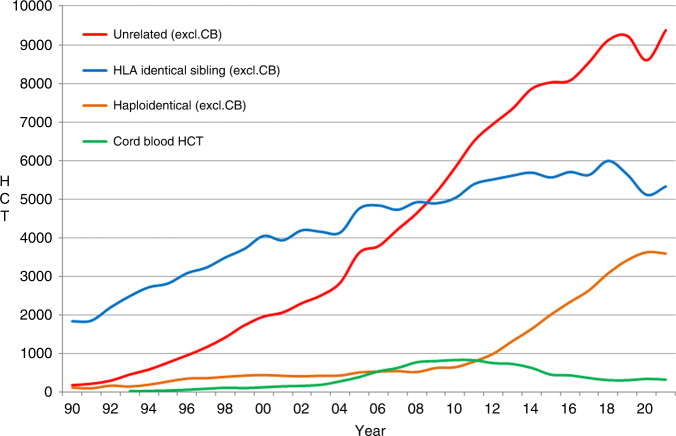
Change in choice of donor type in Europe from 1990 to 2021.

In 2021, changes were reported in the choice of donor (Fig. 4). The overall numbers of patients treated with family donors increased by +2.1% (−3.5% in 2020), however, variation was seen within the choice of family donor used. HLA identical sibling and syngeneic twin donors increased by +4.2% (−9.3% in 2020), but the increase observed in haploidentical donors of +6.2% in 2020, decreased by −1.2% in 2021. For unrelated donors an increase of +9% compared to the decrease of −6.8% in 2020 was seen. In 2020, we observed for the first time in several years that the rate of cord blood HCT for all donor types increased by +11.7% from 309 to 345 and mainly included unrelated cord blood (86%). However, in 2021, a decrease of −5.8% to 325 HCT was observed. Regarding stem cell source, sibling donors receiving either peripheral blood and bone marrow stem cells increased by +4.3% and +4.5% respectively after the decrease seen previously in 2020 of −7% and −16%. However, in haploidentical donors, a small decrease of −1.3% was seen in the use of stem cells harvested from peripheral blood when compared to the increase of +11.6% observed in 2020. Bone marrow stem cells increased by +0.6% compared to the decrease of −12.4% in 2020. In unrelated donor transplants, the use of bone marrow increased once again by +18.9% when compared to the large decrease of −37% observed in 2020. The shift in allogeneic HCT from marrow towards peripheral blood as stem cell source observed in 2020 (−16% for sibling donors, −37% for unrelated donors and −13% for haploidentical donors) was no longer observed in 2021, where each increased by +4.5%, +18.9% and +0.6% respectively.

### Changes in autologous HCT 2020 to 2021

The decrease in activity in autologous HCT seen due to the pandemic in the majority of disease indications in 2020 have mostly increased again in 2021. Figure 3b shows the percentage difference in HCT activity reported by indication between the years 2019 and 2020 (left) and between the years 2020 and 2021 (right). The overall increase in all autologous HCT being +3.9% compared to −7.5 in 2020. The main indications for autologous HCT were lymphoid malignancies (90%) with PCD comprising 55% of all autologous HCT patients. In lymphoproliferative disorders the differences between 2021 and 2020 were; for PCD (+4.8% compared to −6.8 in 2020), NHL (+4.3% compared to −8.9%), for HL +7.5% compared to −2.3% and for ALL +3.7% compared to −18.2%. However, for all types of AML the decrease in activity has continued in 2021 by −4.5% (−6.3% in 2020). In solid tumors, the numbers decreased slightly from 1686 in 2020 to 1635 (−3%). For autoimmune diseases, the overall decrease of −44.7% from 539 to 298 seen in 2020 has recovered in 2021 with an increase of +57% to 468 HCT, the majority of which were given for multiple sclerosis (*n* = 388). Although the numbers are still lower than in 2019, the decrease seen followed by an increase was likely related to the pandemic and reflected the EBMT guidelines for transplant recommendations for autoimmune disease specifically developed to manage HSCT delivery and patients during the pandemic phase [28, 29].

### Changes during the pandemic

**Fig. 5:**
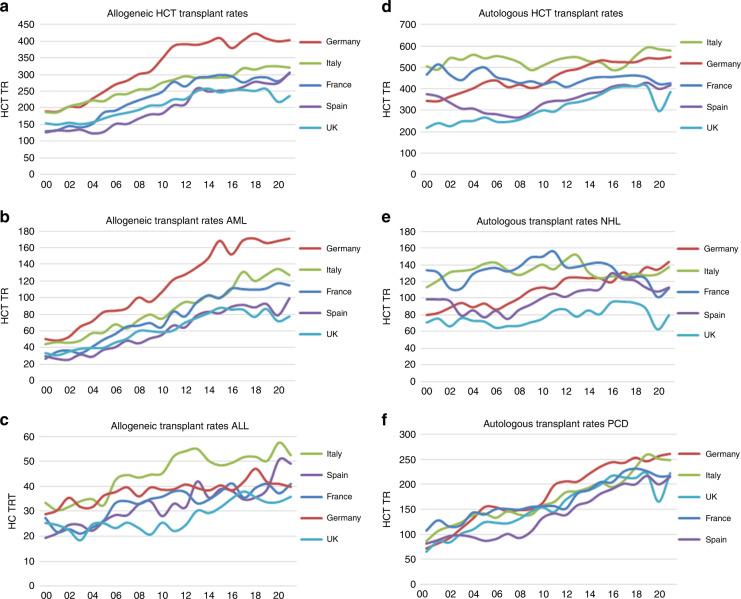
Trend in the transplant rates of HCT for five populous countries in the years 2000 to 2021. **a** Allogeneic HCT (all transplants). **b** Allogeneic HCT for AML (first transplants). **c** Allogeneic HCT for ALL (first transplants). **d** Autologous HCT (all transplants). **e** Autologous HCT for NHL (first transplants). **f** Autologous HCT for PCD (first transplants).

Even though the pandemic continued throughout Europe in 2021, an overall increase in transplant activity of +4.5% was seen when compared to 2020 [28–33]. The use of CAR-T cell technology continued to increase by +35% in 2021 (+65% in 2020). Figure 5a–f show transplant rates for allogeneic (Fig. 5a) and autologous HCT (Fig. 5d) since 2000 and for the main indications AML (Fig. 5b) and ALL (Fig. 5b) for allogeneic HCT and NHL (Fig. 5e) and plasma cell disorders (Fig. 5f) for autologous HCT in five populous countries. It is obvious that the SARS-CoV-2 pandemic did not affect the number of transplanted patients in the same way in all countries with e.g., the UK showing a substantial drop followed by recovery (potentially influenced by coordinated national guidance and policy [29], whereas in other countries changes were minimal. This drop was not uniform, affected autologous HCT more than allogeneic and among autologous more for myeloma than for NHL; in contrast there was a drop for autologous HCT in France for NHL but not for myeloma.

### Changes in transplant rates 2020 to 2021

Assessing transplant rates per 10 million population (TR) allowed the comparison of activity in countries adjusted for differences in population size. In the 2021 survey, the TR rates for allogeneic HCT within European countries only, ranged from 5.4 in Georgia and 11.6 in Ukraine to 403.7 in Germany and 435.7 in Israel (median number of HCT 106 and TR 144.6). Five countries did not report any allogeneic HCT (Bosnia and Herzegovina, Cyprus, Iceland, Latvia and Luxembourg). For autologous HCT, rates ranged from 1.0 in Azerbaijan to 580.6 in Italy (median number of HCT 160 and TR 227.8). All countries participating in the annual survey reported doing autologous HCT.

Comparing the transplant rates in 2021 with those reported in 2020, differences were seen with the 50 countries that reported in both years. Within allogeneic HCT, the TR increased in 31 countries, in 10 countries they continued to go down and in a further 9 countries, no major change was seen. For autologous HCT, an increased in TR was observed in 23 countries, a continued decrease in 19 countries and in 8 countries there was no major change seen. For instance for allogeneic HSCT the transplant rates dropped in Norway from 303/per 10 Million (Mio) inhabitants to 272 (decrease by 31/per 10 Mio) whereas in Lithuania the rate increased from 211 to 264 (increase by 53/per 10 Mio) and in Israel from 393 to 436 (increase by 43/per 10 Mio). For autologous HCT the largest drop was in Croatia (467 to 399 by 68 transplants/per 10 Mio), while in the UK, the transplant rate went from 297 to 387, an increase by 90 transplants/10 Mio. How much is due to the continuing SARS-CoV-2 pandemic or change in activity due to other treatment protocols is unknown.

### Cellular therapy

Table 2 shows the number of patients who received advanced cellular therapy and DLI performed in 2021. Un-manipulated DLIs were reported in 3245 patients, which is an increase of +6.2% compared to 2020. The majority of DLIs were given for relapse (*n* = 1337) and graft enhancement/failure (*n* = 848).

In addition, a total of 3494 patients (15.4% increase) in 288 centers from 31 countries received other forms of hematopoietic cellular therapies that qualified as medicinal products rather than cell transplants [16]. In 2021, the most remarkable increase seen again was in gene-modified T cells, notably CAR-T cells, increasing from 1874 in 2020 to 2524 in 2021 (+34.7%).

**Fig. 6:**
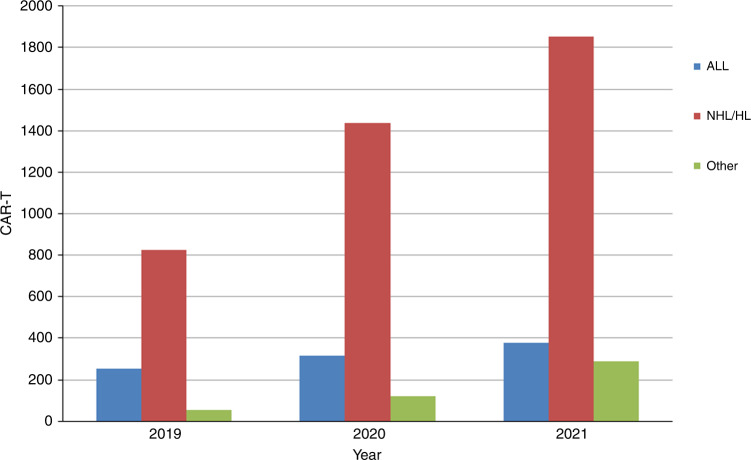
Absolute number of patients receiving a CAR-T cellular therapy by indication in Europe 2019–2021.

The numbers of patients treated with a CAR-T cell infusions has increased constantly since 2017 regardless of the observations seen in HCT activity due to the SARS-CoV-2 pandemic One hundred and ninety-seven centers in 24 countries reported 2524 CAR-T cellular therapies in 2021. The main indication was lymphoma (*n* = 1851; 99% autologous), followed by ALL (*n* = 379; 89% autologous), other malignancies i.e., myeloma, AID (*n* = 294; 95% autologous) (Fig. 6). The second most widely used cellular therapy other than CAR-T cells in 2021 was mesenchymal stromal cells (*n* = 349; 97% allogeneic, −18% when compared to 2020), their use being mainly to treat graft-versus-host disease [22]. Numbers of other cellular therapy products have not greatly changed since 2019. Specific data on tumor infiltrating lymphocytes is not collected in the annual survey.

**Fig. 7:**
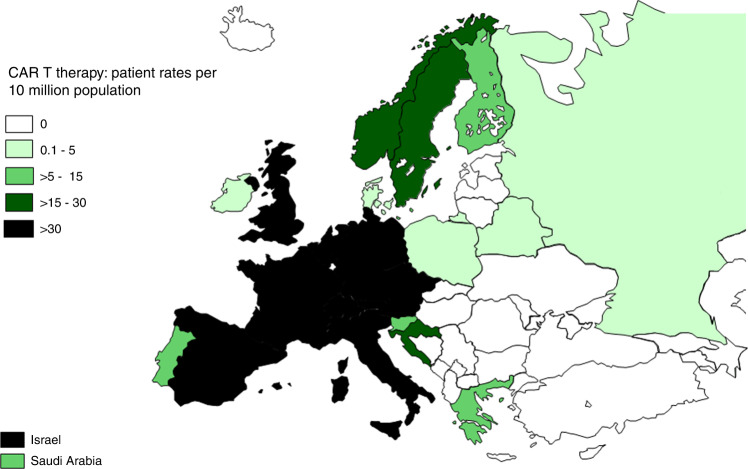
Patient rates per 10 million population for CAR-T cellular therapies in Europe 2021.

CAR-T rates per 10 million population in 2021 ranged from 2.0 in Ireland to 142 in Israel (Fig. 7). The median number of CAR-T patients was 30 and TR 29.3. Fifty-eight patients with allogeneic CAR-T were reported in nine countries. For autologous CAR-T, 24 countries reported 2466 CAR-T’s with a median of 25.5. To analyze whether CAR-T treatment was replacing autologous or allogeneic HCT we calculated CAR-T rates, autologous HCT rates, and allogeneic HCT rates for NHL per country and correlated these.

**Fig. 8:**
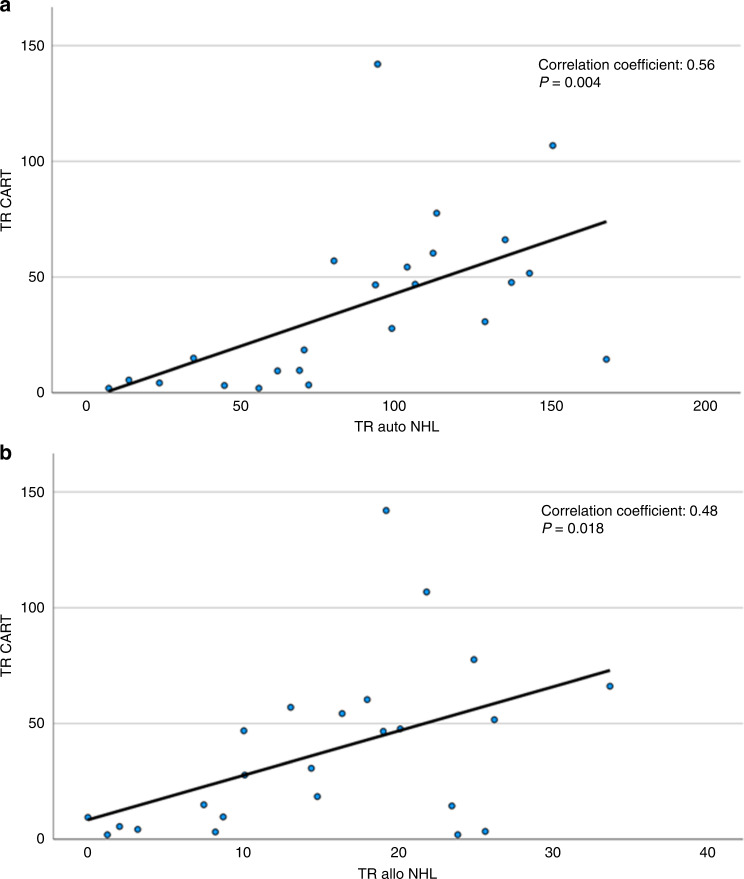
Comparison of rates per 10 million population of allogeneic or autologous HCT in NHL versus CAR-T. **a** Rates per 10 million population of autologous HCT for NHL versus CAR-T rates. **b** Rates per 10 million population of allogeneic HCT for NHL versus CAR-T rates.

If use of CAR-T was to increase at the expense of HCT for NHL, autologous or allogeneic, a negative correlation in the treatment rates would be expected (the higher the rate of CAR-T the lower the rate of HCT for NHL) [34, 35]. Here we find by linear regression a significantly positive correlation as shown in Fig. 8a, b with correlation coefficients of 0.56 for autologous *P* = 0.004 and 0.48 *P* = 0.018 for allogeneic HCT with CAR-T treatment rates per country. For this analysis, only the 24 countries with reported CAR-T treatments were used. CAR-T treatment rates correlate with the wealth of a country, with median CAR-T rate being 6.98 in countries with a GNI < 40′000 USD and 47.3 in countries with a GNI > 40’000 USD *p* = 0.014. We also looked at rates of allogeneic and autologous HCT for NHL in the same countries; there was a significant difference *P* = 0.014 between countries with high GNI and with very high GNI (transplant rates 7.8 vs. 19.1/10 Mio) for allogeneic HCT but not for autologous HCT *P* = 0.16 (transplant rates 39.5 vs. 104.8/10 Mio) [36].

## Discussion

The EBMT activity survey has been conducted annually since 1990 [1]. Over 47,000 transplants in more than 43,000 patients were reported in 2021. The largest number of transplants ever reported was in 2019 (48,412 in 43,581 patients). The decrease observed in transplant activity in 2020 likely due to the SARS-CoV-2 pandemic did not continue in 2021 despite the ongoing pandemic issues. Absolute numbers in 2021 (47,412 in 43,109 patients) almost reached those reported pre pandemic in 2019 (48,412 in 43,581 patients) thus showing partial recovery after the drop associated with SARS-CoV-2. However, the expected annual increase over the years of approximately 5% cannot be seen during this time period. The use of DLI increases continuously over the last 20 years (Fig. 1b) and almost tripled over this time period. Why DLI increased somewhat more than allogeneic HCT and why a pandemic associated drop appears not to be seen, is unclear. Possible explanations include the greater ease in collecting DLI compared to hematopoietic stem cells. Other changes observed in the first year of the pandemic significant or not were reversed in the second year: a drop of marrow transplants for marrow failure saw an increase; the increased use of cord blood saw a drop; decreasing numbers of unrelated donor HCT was reversed; transplant for CML decreased and increased again; re-starting activity in autoimmune diseases was observed. Some of these changes are very likely pandemic related, reflecting the difficulties organizing unrelated donor transplants, hence using other donors and cord blood, as well as postponing or replacing HCT for less urgent indications. The shift from marrow use to peripheral blood and back is also likely related to availability of operating theaters for marrow harvest during the first year of the pandemic. Evolution of SARS-CoV-2 variants, vaccination and new treatments available for SARS-CoV-2 infection may have potentially contributed to the recovery of HCT activity in all indications. Variation in HCT activity between five populous countries (France, Germany, Italy, Spain, UK), with arguably similar economic status, was noted across the last two decades, and also specifically within the Covid-19 pandemic.

We see an impressive increase in the use of CAR-T cell treatments regardless of the pandemic. Whether this increase of +35% would have been more impressive without the pandemic is difficult to assess. This increase is mainly in wealthy countries with very high GNI. At the same time, we attempted to delineate whether CAR-T was used instead of HCT and found positive correlations for autologous and allogeneic HCT for NHL with CAR-T treatments indicating that again, access to such care was higher in very high-income countries. It is not surprising that this very expensive treatment tends to be used in very high-income countries and that access to treatment is not uniform across Europe.

We had previously speculated that development of innovative treatments, CAR-T cellular therapy, bispecific and armed antibodies for myeloma and NHL would result in decreasing use of HCT technology. The partial recovery of HCT rates in the second year of the pandemic does not speak in favor of a massive shift in this direction as yet.

The annual activity survey of the EBMT reflects current activity and trends in the field of transplant technology. We showed partial recovery after the decrease of transplant activity in 2020. The impressive continuous increase in CAR-T cell activity reflects the wide adoption of a newly approved modality supported by academic activities. We show that this expensive new technology is developing almost exclusively in high-income countries.

Despite EBMT recommendations for indications for transplant aiming to standardize practice [4], there appears to be no ‘ideal’ transplant activity rate across countries, even with similar economies. Ongoing studies using the EBMT benchmarking model with registry and survey data aim to assess the impact during the Covid-19 pandemic and, more generally, the impact of international variation in activity and clinical practice across countries with similar and variable economies on survival outcomes.

In summary, this report is valuable for the dissemination of the most recent information on indications, donor and stem cell usage and benchmarking, which will ultimately be beneficial in health care planning.

## Supplementary information

Supplementary file_Appendix EBMT activity survey 2021

## Data Availability

Datasets may be available upon request via EBMT Partnering (partnering@ebmt.org).
