# Supplementary material for: Hematopoietic cell transplantation and cellular therapies in Europe 2021. The second year of the SARS-CoV-2 pandemic. A Report from the EBMT Activity Survey
Source: Bone Marrow Transplant. 2023 Mar 6;58(6):647–58. doi: 10.1038/s41409-023-01943-3 (PMC9987384; doi:10.1038/s41409-023-01943-3)
Supplement: Supplementary file 1 — Supplementary file_Appendix EBMT activity survey 2021 [file 41409_2023_1943_MOESM1_ESM.pdf]

APPENDIX 2021

EBMT Transplant Activity Survey: List of reporting transplant centers in 2021

(Number of reporting centers: total 1st HCT (total all HCT) N allogeneic 1st HCT / N autologous 1st HCT)

Algeria: (2 teams: 233 (234) 158/75)
Alger, Centre Pierre et Marie Curie, ads, peds, CIC 703, R. Hamladji (199 (200) 146/53)
Oran, University Hospital of Oran, ads, CIC 993, M. Bekadja (34 (34) 12/22)

Armenia: (1 team: 23 (23) 4/19)
Yerevan, Hematology Centre R. H. Yeolyan, ads, peds, CIC 891, K. Meliksetyan (23 (23) 4/19)

Austria: (13 teams: 595 (657) 265/330)
Graz, Universitäts Kinderklinik, peds, CIC 593, C. Urban, W. Schwinger (10 (10) 4/6)
Graz, University of Graz, ads, CIC 308, H. Greinix, H. Sill (92 (102) 43/49)
Innsbruck, University Hospital, ads, peds, CIC 271, D. Wolf, D. Nachbaur (79 (85) 34/45)
Klagenfurt, Klinikum Klagenfurt, ads, CIC 0, S. Eder (14 (15) 0/14)
Linz, AOKH der Elisabethinen, ads, CIC 594, J. Clausen, A. Weltermann (108 (124) 62/46)
Linz, Kepler University Hospital, ads, CIC 343, C. Schimitt (4 (5) 0/4)
Salzburg, LKA Salzburg, ads, peds, CIC 356, R. Greil, G. Rass (32 (33) 0/32)
St. Pölten, University Hospital St. Pölten, ads, CIC 474, G. Krajnik, Ch. Fertl (17 (18) 0/17)
Vienna, Donauspital, ads, CIC 767, C. Sebesta, P. Kier (11 (17) 0/11)
Vienna, Hanusch- Krankenhaus, ads, CIC 743, F. Keil (34 (38) 0/34)
Vienna, Kilinik Ottakring, Wilhelminenspital, ads, CIC 828, W. Hilbe, N. Zojer (35 (42) 0/35)
Vienna, St. Anna Kinderspital, peds, CIC 528, H. Pichler,W. Holter, S. Karlhuber (28 (37) 21/7)
Vienna, Universitätsklinik fur Innere Medizin-AKH, ads, CIC 227, W. Rabitsch (131 (131) 101/30)

Azerbaijan: (1 team: 16 (16) 15/1)
Baku, Republican Thalassemia Centre, ads, peds, CIC 176, N. Aliyeva Rafiq, S. Saila Rauf (16 (16) 15/1)

Republic of Belarus: (2 teams: 173 (179) 53/120)
Minsk, Belorussian Center, peds, CIC 591, O. Aleinikova, Y. Mareika (43 (49) 32/11)
Minsk, Scientific Centre of Surgery, Transplantology and Hematolgy, ads, CIC 0, N. Milanovich (130 (130) 21/109)

Belgium: (17 teams: 851 (911) 375/476)
Antwerp, Stuivenberg ZH, ads, CIC 339, W. Ka Lung (23 (23) 6/17)
Antwerp, University Antwerpen, ads, CIC 996, A. Gadisseur (58 (60) 27/31)
Brugge, A.Z. St. Jan, ads, CIC 506, D.Selleslag, J.v.Droogenbroeck (78 (79) 37/41)
Brussels, Clinique Universitaire St. Luc, ads, peds, CIC 234, X. Poiré, C. Vermylen (78 (81) 49/29)
Brussels, Institute Jules Bordet and the Childrens Hospital, ads, peds, CIC 215, N. Meuleman, C. Devalck (81 (83) 34/47)
Brussels, U.L.B. Hôpital Erasme, ads, CIC 0, V. De Wilde (12 (12) 0/12)
Brussels, University Hospital, ads, peds, CIC 630, R. Schots, F. Trullemans (40 (44) 20/20)
Charleroi, Grand Hôpital de Charleroi Notre-Dame, ads, CIC 349, D. Pranger (9 (11) 0/9)
Gent, University Hospital, ads, peds, CIC 744, C. Dhooge, T. Kerre, V. Bordon (104 (109) 49/55)
Haine St. Paul, Hôpital de Jolimont, ads, CIC 2343, H. Petre, F. Van Obbergh (15 (16) 0/15)
Hasselt, Jessa Ziekenhuis, ads, CIC 632, K. Theunissen (50 (56) 0/50)
Leuven, University Hospital Gasthuisberg and Leuven, ads, peds, CIC 209, J. Maertens,G.Verhoef, A. Uyttebroeck, M. Renard (119 (123) 77/42)
Liège, University Hospital Sart-Tilman, ads, peds, CIC 726, Y. Béguin, B de Prijck (62 (74) 32/30)
Roeselare, AZ Delta, H. Hart Ziekenhuis, ads, CIC 646, D. Deeren (30 (35) 14/16)
Turnhout, AZ Turnhout, ads, CIC 910, I. Vrelust, B. Hodossy (13 (13) 0/13)
Wilrijk-Antwerp, St. Agustinus Hospital, ads, CIC 715, J. Lemmens, C. Schuermans (17 (21) 0/17)
Yvoir, Clinique universitaire de Mont-Godinne, ads, CIC 2342, C. Doyen (62 (71) 30/32)

Bosnia-Herzegovina: (1 team: 30 (30) 0/30)
Sarajevo, Clinical Centre, ads, CIC 198, A. Sofo-Hafizovic (30 (30) 0/30)

Tuzla, University Clinical Centre, (ads), H. Sahovic (no report)

Bulgaria: (2 teams: 88 (93) 35/53)
Sofia, National Centre of Hematology, ads, CIC 859, B. Spassov, G. Arnaudov, K. Simeonova (82 (87) 33/49)
Sofia, University Hospital Queen Johanna-Isul, peds, CIC 346, D. Konstantinov, B. Avramova (6 (6) 2/4)

Croatia: (4 teams: 210 (255) 84/126)
Split, University Hospital Split, ads, CIC 1033, D. Lozic (9 (9) 0/9)
Zagreb, Hospital Merkur, ads, CIC 466, D. Radic-Kristo, V. Zatezalo (34 (45) 0/34)
Zagreb, University Hospital Dubrava, ads, CIC 407, V. Pejsa, O. Jaksic, Z. Prka (13 (13) 0/13)
Zagreb, University Hospital Rebro, ads, peds, CIC 302, R. Vrhovac, R. Serventi-Seiwerth (154 (188) 84/70)

Cyprus: (1 team: 40 (40) 0/40)
Nicosia, Nicosia General Hospital, ads, CIC 575, K. Melanthiou, , C. Stylianou (40 (40) 0/40)

Czech Republic: (9 teams: 580 (701) 227/353)
Brno, Masaryk University Hospital, ads, peds, CIC 597, J. Mayer, Z. Racil, J. Sterba (108 (121) 29/79)
Hradec Kralové, Charles University Hospital, ads, CIC 729, P. Zak (56 (64) 23/33)
Olomouc, University Hospital, ads, CIC 574, L. Raida (41 (53) 19/22)
Ostrava, Universtity Hospital Ostrava, ads, CIC 156, R. Hajek (50 (63) 19/31)
Pilsen, Charles Hospital, ads, CIC 718, P. Jindra, A. Jungova (126 (151) 48/78)
Prague, Charles University, ads, CIC 745, M. Trneny (63 (84) 0/63)
Prague, Charles University Hospital, ads, CIC 318, T. Kozak (31 (32) 0/31)
Prague, Institute of Hematology and Blood Transfusion, ads, CIC 656, A. Vitek (63 (74) 63/0)
Prague, University Hospital Motol, peds, CIC 452, P. Sedlacek (42 (59) 26/16)

Denmark: (3 teams: 288 (321) 154/134)
Aalborg, Aalborg Hospital, ads, CIC 848, I. Christiansen, J. Baech, K.Nielsen (23 (28) 0/23)
Aarhus, Aarhus Amtssygehus, ads, CIC 634, M. Thorsgaard (113 (127) 55/58)
Copenhagen, Rigshospitalet, ads, peds, CIC 206, H. Sengelov, C. Heilmann, P. Josefsson (152 (166) 99/53)

Estonia: (2 teams: 86 (96) 27/59)
Tallinn, North Estonia Medical Centre, ads, CIC 984, K. Palk (36 (38) 0/36)
Tartu, University Hospital, ads, peds, CIC 746, A.Kaare (50 (58) 27/23)

Finland: (7 teams: 446 (456) 161/285)
Helsinki, Children's Hospital, peds, CIC 219, K. Vettenranta (30 (33) 23/7)
Helsinki, Helsinki University Central Hospital, ads, peds, CIC 515, R. Niittyvuopio (121 (125) 73/48)
Helsinki, Helsinki University Hospital, ads, CIC 833, S. Mannisto (20 (20) 0/20)
Kuopio, University Hospital, ads, peds, CIC 396, A. Partanen (49 (50) 0/49)
Oulu, Oulu University Central Hospital, ads, peds, CIC 690, M. Säily (46 (46) 0/46)
Tampere, University Hospital, ads, peds, CIC 635, M. Sankelo, M. Sinisalo (60 (62) 0/60)
Turku, University Central Hospital, ads, CIC 225, U. Salmenniemi, M. Itäla-Remes (120 (120) 65/55)

France: (73 teams: 4,584 (4,952) 1,943/2,641)
Amiens, CHU d'Amiens, ads, peds, CIC 955, A. Charbonnier, J.P. Marolleau (77 (83) 44/33)
Angers, Centre Hospitalier, ads, peds, CIC 650, M. Hunault-Berger, S. Francois (79 (86) 35/44)
Argenteuil, Hopital Victor Dupouy, ads, CIC 199, A. Al Jijakli (19 (19) 0/19)
Arras, Groupe Hospitalier Artois Ternois, ads, CIC 999, P. Lionne-Huyghe (15 (15) 0/15)
Bayonne, C.H. De la Cote Basque, ads, CIC 0, S. Labarrere, A. Banos (33 (33) 0/33)
Besancon, Hopital Jean Minjoz and St.Jacques, ads, peds, CIC 233, E.Deconinck (75 (76) 46/29)
Bordeaux, CHU Hopitalier Pellegrin- Enfants, peds, CIC 978, C. Jubert, Y. Perel (28 (37) 18/10)
Brest, Hopital Morvan, CHU de Brest, ads, peds, CIC 0, S. Creachcadec, G. Guillerm, C. Berthou (65 (77) 28/37)
Caen, CHU Caen Institut d’hématologie de Basse-Normandie CHU, ads, CIC 251, G. Damaj,S. Chantepie (78 (82) 34/44)
Clermont Ferrand, CRCTCP, CHU Estaing, ads, peds, CIC 273, J.-O. Bay, E. Rochette (91 (105) 34/57)

Colmar, Louis Pasteur Hospital, ads, CIC 0, J. Chapiro (7 (7) 0/7)
Corbeil Essonne, Hopital Gilles de Corbeil, ads, CIC 0, B. Joly, S. Haiat (23 (27) 0/23)
Créteil, Hopital Henri Mondor, ads, CIC 252, C. Cordonnier, S. Maury (36 (38) 36/0)
Créteil, Hopital Henri Mondor, ads, CIC 432, C. Haioun, J. Dupuis (29 (42) 0/29)
Dijon, Hopital des Enfants, ads, CIC 0, D. Caillot (66 (75) 0/66)
Dunkerque, Centre Hospitalier, ads, CIC 0, J-M. Pignon, M. Wetterwald (11 (11) 0/11)
Grenoble, CHV Grenoble Alpes, ads, peds, CIC 270, J.Y. Cahn, C.E. Bulabois (98 (109) 60/38)
La Réunion, CHU Felix Guyon, Saint Denis Centre, ads, CIC 0, S. Vanderbecken (15 (15) 0/15)
La Réunion, St. Pierre CHU Sud La Réunion, ads, CIC 404, C. Mohr (16 (19) 1/15)
Le Chesnay, Hôpital André Mignot, Varsailles, ads, CIC 0, Ph. Rousselot, S. Castaigne (42 (42) 0/42)
Lens, Service d'Hématologie, ads, CIC 0, C. Bories, L. Stalnikiewicz (11 (11) 0/11)
Lille, Centre Hospitalier Saint Vincent, ads, CIC 0, B. Carpentier, L. Pascal (15 (15) 0/15)
Lille, Centre Oscar Lambret, peds, CIC 0, A. Defachelles (11 (16) 0/11)
Lille, Hopital Claude Huriez, ads, CIC 277, I. Yakoub-Agha (142 (149) 107/35)
Lille, Hopital Jeanne de Flandre, peds, CIC 963, B. Bruno, B. Nelken (24 (27) 24/0)
Limoges, CHU Dupuytren, ads, CIC 977, P. Turlure, D. Bordessoule (45 (46) 22/23)
Lyon, Centre Hospitaier Lyon Sud, ads, CIC 671, H. Labussiere-Wallet (73 (80) 71/2)
Lyon, Centre Léon Bérand, ads, CIC 241, E. Nicolas-Virelizier (55 (70) 0/55)
Lyon, Hospices Civils de Lyon, ads, CIC 901, H. Ghesquieres (90 (98) 0/90)
Lyon, Institue d'Hématologie et d'Oncologie Pédiatrique, peds, CIC 806, Y. Bertrand (44 (49) 30/14)
Marseille, Hopital Timone Enfants, peds, CIC 301, G. Michel, C. Coze (39 (44) 28/11)
Marseille, Institut Paoli I. Calmettes, ads, CIC 230, D. Blaise, C. Chabannon (234 (254) 141/93)
Marseille Bouches du Rhone, Centre Hospitalier Universitaire La Conception, ads, CIC 158, R. Costello (17 (17) 0/17)
Meaux, CHU de Meaux, ads, CIC 194, J. Frayfer, L. Fouillard (14 (15) 0/14)
Montpellier, CHR Lapeyronie and CHU Arnaud de Villeneuve, ads, peds, CIC 926, N. Fegueux, A. Sirvent (221 (222) 71/150)
Mulhouse, Hopital E. Muller, ads, CIC 944, B. Drénou, M. Ojeda-Uribe (25 (29) 0/25)
Nantes, CHU Nantes, ads, peds, CIC 253, P. Chevallier, S. Legouill, F. Rialland (200 (219) 88/112)
Nice, Centre Antoine Lacassagne, ads, CIC 973, L. Gastaud (61 (61) 0/61)
Nice, Hopital de l'Archet, ads, peds, CIC 523, P. Rohrlich, T. Cluzeau (59 (62) 45/14)
Orleans, CHR Orléans, ads, CIC 0, M. Alexis, C. Benbrahim (27 (29) 0/27)
Paris, Clarmart, Hopital d'Instruction des Armées Percy, ads, CIC 0, JV. Malfuson (59 (67) 31/28)
Paris, Hôpital Cochin, ads, CIC 0, D. Bouscary (48 (56) 0/48)
Paris, Hopital d'enfants Armand Trousseau, peds, CIC 675, G. Leverger, A. Petit (4 (4) 0/4)
Paris, Hôpital Neckar des enfants malades, peds, CIC 201, B. Neven, S. Blanche (45 (50) 45/0)
Paris, Hôpital Necker, ads, CIC 160, O. Hermine, F. Suarez (88 (91) 50/38)
Paris, Hôpital Pitié Salpétière, ads, CIC 262, V. Leblond, S. Nguyen Quoc (107 (114) 43/64)
Paris, Hôpital Robert Debré, peds, CIC 631, JH. Dalle, A. Baruchel (60 (60) 60/0)
Paris, Hôpital St. Antoine, ads, CIC 775, M. Mohty, S. Lapusan (97 (105) 49/48)
Paris, Hôpital St. Louis, ads, CIC 805, C. Thieblemont (27 (27) 0/27)
Paris, Hôpital St. Louis, ads, peds, CIC 207, G. Socié, M. Robin, R Peffault de La Tour (141 (147) 136/5)
Paris, Hôpital Tenon, ads, CIC 747, J.P. Lotz, F. Selle (5 (11) 0/5)
Paris, Immuno-Hèmatologie, Hôpital St. Louis, ads, CIC 0, B. Arnulf (74 (74) 0/74)
Paris, Institut Curie, peds, CIC 702, Ph. Brault (18 (27) 0/18)
Pessac, Hôpital du Haut Leveque, CHU Bordeaux, ads, CIC 267, N. Milpied,E. Forcade (193 (202) 95/98)
Poitiers, CHU de Poitiers, Hôpital La Miletrie, ads, CIC 264, X. Leleu, M. Maillard, (114 (119) 48/66)
Pontoise, Hospital René Dubos, ads, CIC 0, H. Gonzalez, I. Vaida (21 (21) 0/21)
Reims, Hop. Robert Debré, ads, CIC 959, A. Delmer, C. Himberlin (32 (36) 0/32)
Rennes, CHU Rennes, ads, CIC 6611, T. Lamy, M Bernard (109 (116) 45/64)
Rennes, Clinique Médical Infantil, CHRU, peds, CIC 6612, V. Gandemer (22 (27) 16/6)
Roubaix, Hopital V. Provo, ads, CIC 0, I. Plantier-Colcher (13 (13) 0/13)
Rouen, Centre Henri Becquerel, ads, CIC 941, H. Tilly, N. Contentin (83 (92) 28/55)
Rouen, Hopital Charles Nicolle, peds, CIC 0, P. Schneider, N. Buchbinder (16 (22) 9/7)
Saint Priest en Jarez, Institut de Cancérologie Lucien Neuwirth, ads, CIC 250, D. Guyotat, J. Cornillon (49 (52) 25/24)
Saint Quentin, Centre Hospitalier De Saint Quentin, ads, CIC 406, R. Garidi (17 (17) 0/17)
St. Cloud, Institut Curie, Hopital René Huguenin, ads, CIC 0, S. Glaisner (49 (60) 0/49)
Strasbourg, ICANS, ads, peds, CIC 672, B. Lioure, L. Fornecker, C. Paillard (133 (136) 58/75)

Toulouse, Hopital Purpan, peds, CIC 0, H. Rubie, G. Alphonsa (5 (7) 0/5)
Toulouse, Institut Universitaire du Cancer Toulouse Oncopole, ads, CIC 624, A. Huynh, C. Recher (200 (202) 80/120)
Tours, Hôpital Bretonneau, ads, peds, CIC 272, E. Gyan, L. Sutton (104 (105) 15/89)
Troyes, Centre Hospitalier de Troyes, ads, CIC 472, A. Santagostino (22 (22) 0/22)
Vandoeuvre-les-Nancy, Hôpital d'Enfants and Brabois, ads, peds, CIC 676, M. Rubio, P. Feugier, A. Campidelli, C. Pochon (125 (136) 69/56)
Valenciennes, Centre Hosp. de Valenciennes, (ads), M. Simon, N. Cambier (no report)
Villejuif, Gustave Roussy Cancer Campus, ads, peds, CIC 666, J-H. Bourhis, C. Castilla-Llorente (163 (184) 78/85)
Villejuif, Institut Gustave Roussy, peds, CIC 503, D. Valteau-Couanet (31 (39) 0/31)

Georgia: (1 team: 47 (47) 2/45)
Tbilisi, High Technology Medical Center, ads, CIC 471, G. Ingorokva (47 (47) 2/45)

Germany: (117 teams: 6,859 (7942) 3,095/3,764)
Aachen, Universitätsklinikum, ads, CIC 0, T. Brümmendorf, E. Jost (96 (98) 50/46)
Aachen, Universitätsklinikum, peds, CIC 348, U. Kontny (8 (8) 7/1)
Augsburg, Klinikum Augsburg, ads, CIC 152, C. Schmid (76 (90) 41/35)
Bad Saarow, Humaine Klinikum, ads, CIC 0, R. Ratei (19 (25) 0/19)
Bamberg, Klinikum am Bruderwald, ads, CIC 0, R. Seggewiss-Bernhardt (18 (30) 0/18)
Berlin, Charite, Campus Virchow Klinikum, ads, CIC 807, I. Blau (212 (259) 105/107)
Berlin, Charité, Campus Virchow Klinikum, peds, CIC 336, J. Schulte (34 (40) 27/7)
Berlin, HELIOS Klinikum Berlin Buch, ads, peds, CIC 518, H. Baurmann (115 (120) 59/56)
Berlin, Vivantes Klinikum Neukoelln, ads, CIC 105, M de Wit. L. Marretta (35 (52) 0/35)
Bielefeld, Evangelisches Klinikum Bethel, ads, CIC 116, F. Weissinger (13 (16) 0/13)
Bielefeld, Klinikum Bielefeld, ads, CIC 949, M. Görner (12 (13) 0/12)
Bochum, Knappschaftskrankenhaus, ads, CIC 124, R. Schroers (121 (151) 47/74)
Bonn, Johanniter-Krankenhaus und Waldkrankenhaus, ads, CIC 0, C. Sippel (35 (50) 0/35)
Bonn, Universitätsklinikum, ads, CIC 134, P. Brossart (74 (80) 38/36)
Bonn, Universitätsklinikum, peds, CIC 403, D. Dilloo (6 (6) 4/2)
Braunschweig, Städtisches Klinikum, ads, CIC 674, J. Krauter (47 (58) 0/47)
Bremen, Evangelistisch Diakonie-Krankenhaus GmbH, ads, CIC 111, R.U.Trappe, N. Winkelmann (21 (25) 0/21)
Bremen, Klinikum Bremen-Mitte, ads, CIC 602, B.Hertenstein (48 (57) 18/30)
Chemnitz, Klinikum Chemnitz GmbH, ads, CIC 104, M. Hänel, A.Morgner (57 (68) 17/40)
Cottbus, Carl-Thiem-Klinikum, ads, CIC 102, M. Schmidt-Hieber (10 (13) 0/10)
Dortmund, St. Johannes Hospital, ads, CIC 125, R. Meyer, M.Hindahl (44 (49) 18/26)
Dresden, Universitätsklinikum Carl Gustav Carus, ads, CIC 808, M. Bornhäuser, J. Schetelig (147 (173) 85/62)
Dresden, Universitätsklinikum Carl Gustav Carus, peds, CIC 808, J. Hauer (9 (9) 7/2)
Duisburg, HELIOS Klinikum, ads, CIC 519, C. Aul (47 (52) 24/23)
Düsseldorf, Heinrich Heine Universitätsklinikum, ads, CIC 390, G. Kobbe (122 (153) 64/58)
Düsseldorf, Universitätsklinikum, peds, CIC 651, A. Borkhardt, R.Meisel (27 (33) 20/7)
Erfurt, Helios-Klinikum, ads, CIC 966, H. Sayer, V. Schmidt (40 (42) 0/40)
Erlangen, Universitäts Klinik für Kinder und Jugendliche, peds, CIC 809, K. D. Stachel (10 (12) 7/3)
Erlangen, Universitätsklinikum, ads, CIC 809, A. Mackensen, W. Rösler, J. Winkler (93 (96) 50/43)
Essen, Evangelisches Krankenhaus Essen-Werden GmbH, ads, CIC 784, P.Reimer, M. Wattad (61 (65) 16/45)
Essen, Universitätsklinikum, ads, CIC 259, D.W. Beelen, H. Ottinger (196 (213) 112/84)
Essen, Universitätsklinikum, peds, CIC 259, R. Beier, O. Basu (17 (19) 12/5)
Essen, West German Cancer Center, ads, CIC 0, M. Schuler, S. Bauer (9 (9) 0/9)
Flensburg, St. Franziskus Hospital, ads, peds, CIC 970, N. Basara, H. Menzel (28 (30) 13/15)
Frankfurt, J. W. Goethe Universität, peds, CIC 138, T. Klingebiel, P.Bader (38 (40) 35/3)
Frankfurt, Klinikum Frankfurt Oder, ads, CIC 190, M. Kiehl (37 (42) 25/12)
Frankfurt, Krankenhaus Bethanien, ads, CIC 193, W. Knauf (25 (36) 0/25)
Frankfurt, Universitätsklinikum d. J. W. Goethe, ads, CIC 297, H. Martin, H. Serve (119 (131) 68/51)
Freiburg, Universitatsklinikum, peds, CIC 810, C. Niemeyer, B. Strahm (27 (28) 23/4)
Freiburg, Universitätsklinikum, ads, CIC 810, J.Duyster, J. Finke, M.Engelhardt (227 (245) 87/140)

Georgsmarienhütte, Niels Stensen Kliniken Franziskus Hosptital Hardenberg, ads, CIC 0, R. Peceny (13 (17) 0/13)
Giessen, Universitätsklinikum, ads, CIC 463, A. Burchardt, M. Rummel (17 (19) 0/17)
Giessen, Universitätsklinikum, peds, CIC 326, C. Mauz-Körholz (14 (14) 11/3)
Göttingen, Universitätsklinikum, ads, CIC 552, G. Wulf, L. Trümper (170 (190) 82/88)
Greifswald, Universitätsklinikum, ads, CIC 530, W. Krüger (59 (73) 32/27)
Greifswald, Universitätsklinikum, peds, CIC 908, H. Lode (6 (7) 5/1)
Hagen, St. Marien Hospital, ads, CIC 536, H. W. Lindemann (20 (31) 0/20)
Halle, Universitätsklinikum, ads, CIC 338, L. Müller,T.Weber (85 (103) 39/46)
Halle, Universitätsklinikum, peds, CIC 654, J. Höll, K.Kafa (8 (8) 6/2)
Hamburg, Asklepios Klinik Altona, ads, CIC 366, H. Salwender (26 (36) 0/26)
Hamburg, Asklepios Klinik St. Georg, ads, CIC 153, A. Elmaagacli (72 (74) 43/29)
Hamburg, Universitätsklinikum Eppendorf, ads, CIC 614, N.Kröger (169 (195) 151/18)
Hamburg, Universitätsklinikum Eppendorf - Onkologisches Zentrum, ads, CIC 673, C. Bokemeyer (116 (129) 0/116)
Hamburg, Unversitätsklinikum Eppendorf, peds, CIC 882, I. Müller (44 (51) 43/1)
Hamm, Evangelisches Krankenhaus, ads, CIC 509, E.Lange (12 (13) 0/12)
Hamm, St. Barbara-Klinik, ads, CIC 470, H. Duerk (24 (28) 0/24)
Hannover, Klinikum Siloah, ads, CIC 342, K. Marienhagen (22 (34) 0/22)
Hannover, Medizinische Hochschule, ads, CIC 295, A. Ganser (135 (146) 98/37)
Hannover, Medizinische Hochschule, peds, CIC 295, C. Kratz, KW. Sykora (31 (34) 25/6)
Heidelberg, Angelika Lautenschläger-Klinik, peds, CIC 524, A. Kulozik, J. Greil (9 (9) 8/1)
Heidelberg, Universitätsklinikum, ads, CIC 524, C. Müller-Tidow, P. Dreger (267 (334) 108/159)
Homburg/Saar, Universität des Saarlandes, ads, CIC 7851, N. Bittenbring (74 (94) 27/47)
Homburg/Saar, Universität des Saarlandes, peds, CIC 7852, N. Graf, T. Krenn (1 (1) 0/1)
Jena, FS Universitäts-Kinderklinik, peds, CIC 750, J. Beck, B. Gruhn (9 (10) 7/2)
Jena, Universitätklinikum, ads, CIC 533, I. Higendorf, A. Hochhaus (88 (93) 45/43)
Kaiserslautern, Westpfalzklinikum, ads, CIC 357, G. Held (15 (18) 0/15)
Karlsruhe, Städtische Klinik, ads, CIC 290, M.Bentz, M. Ringhoffer (49 (57) 28/21)
Kassel, Klinikum Kassel, ads, peds, CIC 0, M. Wolf, E. Steinhauer, M. Nathrath (24 (28) 0/24)
Kiel, Städtisches Krankenhaus, ads, CIC 895, R. Repp (25 (29) 0/25)
Kiel, Universitätsklinikum Schleswig-Holstein, ads, CIC 256, M.Gramatzki, T. Valerius (68 (84) 39/29)
Kiel, Universitätsklinikum Schleswig-Holstein, (peds), CIC 256, M. Schrappe (no report)
Koblenz, Stiftungsklinikum Mittelrhein, ads, CIC 879, D.Niemann (28 (28) 0/28)
Köln, Universitätsklinikum, ads, peds, CIC 534, M. Hallek,Ch. Scheid (194 (218) 74/120)
Leipzig, Universitätsklinikum, ads, CIC 389, U. Platzbecker (142 (156) 72/70)
Leipzig, Universitätsklinikum, peds, CIC 389, H. Christiansen (11 (11) 10/1)
Lemgo, Klinikum Lippe, ads, CIC 0, F. Hartmann C.Constantin (17 (24) 0/17)
Lübeck, Universitätsklinikum Schleswig Holstein, ads, CIC 3671, F. Wortmann (53 (59) 23/30)
Lübeck, Universitätsklinikum Schleswig Holstein, peds, CIC 3672, M. Lauten (2 (2) 0/2)
Ludwigshafen, klinikum der Stadt, ads, CIC 140, M.Hoffmann (12 (14) 0/12)
Magdeburg, Universitätsklinikum, ads, CIC 359, T. Heinicker,R. Bartsch (52 (58) 19/33)
Mainz, Universitätsklinikum, ads, CIC 786, E. Wagner-Drouet, M. Theobald (183 (203) 109/74)
Mannheim, Universitätsklinikum, ads, CIC 142, W K. Hofmann, S. Klein (42 (50) 25/17)
Marburg, Philipps Universitätsklinikum, ads, CIC 645, A. Neubauer, A. Burchert (96 (105) 58/38)
Meschede, Klinikum Hochsauerland GmbH, ads, CIC 489, M. Wattad, E. Lange (14 (15) 0/14)
Minden/Westfalen, Klinikum Minden, ads, CIC 113, H. Tischler (35 (47) 0/35)
Möchengladbach, Klinikum Maria Hilf ll, KH St. Franziska, ads, CIC 120, U.Graeven (0 (0) 0/0)
Munich, Haunersches Kinderspital Klinikum Grosshadern, peds, CIC 513, M. Albert (27 (36) 21/6)
Munich, Klinikum Rechts der Isar, ads, CIC 558, M. Verbeek (89 (116) 46/43)
Munich, Klinikum Schwabing, ads, CIC 151, A. Hausmann (52 (56) 33/19)
Munich, Klinikum Schwabing, peds, CIC 189, S.Burdach, A.Wawer (8 (13) 4/4)
Munich, Rotkreuz Klinikum, ads, CIC 883, M. Hentrich (71 (105) 0/71)
Munich, Universitätsklinikum Grosshadern, ads, CIC 513, J.Tischer (94 (97) 52/42)
Münster, Universitätklinikum, ads, CIC 680, M. Stelljes (251 (281) 135/116)
Münster, Universitätklinikum Münster, peds, CIC 505, C. Rössig (29 (37) 23/6)
Nürnberg, Klinikum Nuernberg, ads, CIC 625, M.Wilhelm, K. Schäfer-Eckart (69 (75) 39/30)
Oldenburg, Universitätsklinikum, ads, CIC 749, B. Metzner, CH.Köhne, J. Caspar (126 (126) 28/98)
Osnabrück, Klinikum Osnabrück, ads, CIC 101, C. Petz, R. Peceny (25 (27) 0/25)
Potsdam, Klinikum Ernst von Bergmann, ads, CIC 106, K. Jordan (21 (27) 0/21)

Regensburg, Universitätsklinikum, ads, CIC 787, E. Holler (167 (178) 83/84)
Regensburg, Universitätsklinikum, peds, CIC 787, S. Corbacioglu (20 (20) 18/2)
Rostock, Universitätsklinikum, ads, CIC 585, C. Junghanss (43 (54) 20/23)
Schwäbisch Hall, Diakonie Hospital, ads, CIC 0, L.Jahn (11 (14) 0/11)
Schwerin, Helios Klinik Schwerin, ads, CIC 447, A. Günther (19 (23) 0/19)
Siegen, St. Marien-Krankenhaus, ads, CIC 135, R. Naumann (29 (40) 0/29)
Stuttgart, Diakonissen Krankenhaus, ads, CIC 146, J. Greiner, S. Von Harsdorf (54 (75) 23/31)
Stuttgart, Klinikum Stuttgart, Katharinenhospital, ads, CIC 143, G. Illerhaus (35 (37) 13/22)
Stuttgart, Robert Bosch Krankenhaus, ads, CIC 145, S. Martin, W. Aulitzky, M.Kaufmann (66 (75) 32/34)
Stuttgart, Universitätsklinikum Olgahospital, peds, CIC 701, S. Bielack, E. Koscielniak (1 (1) 0/1)
Tübingen, Universitätsklinikum, ads, CIC 223, L. Kanz, C.Faul (116 (136) 57/59)
Tübingen, Universitätsklinikum, peds, CIC 535, P.Lang (39 (44) 28/11)
Ulm, Kinderklinik Universitätsklinikum, peds, CIC 204, A.Schulz (18 (20) 16/2)
Ulm, Universitätsklinikum, ads, CIC 204, D. Bunjes, H. Döhner (155 (187) 76/79)
Villingen, Schwarzwald-Baar Klinikum, ads, CIC 155, P. La Rosée (21 (24) 0/21)
Wiesbaden, Dr. Horst Schmidt Klinikum, ads, CIC 586, N. Frickhofen, A. Brecht (23 (26) 0/23)
Winnenden, Rems-Murr Klinikum, ads, CIC 180, M. Schaich (23 (30) 0/23)
Wümme, Diakoniekrankenhaus Rotenburg, ads, CIC 871, F.Heits (17 (18) 0/17)
Würzburg, Universitätsklinikum, ads, CIC 712, H.Einsele (194 (241) 72/122)
Würzburg, Universitätsklinikum, peds, CIC 196, P. Schlegel (13 (18) 10/3)

Greece: (12 teams: 360 (390) 216/144)
Alexandroupolis, Thrace University Med. School, ads, CIC 0, I. Kotsianidis (0 (0) 0/0)
Athens, Aghia Sophia Childrens Hospital, peds, CIC 752, V. Kitra-Roussos (59 (66) 46/13)
Athens, Athens Medical Center, ads, CIC 0, A. Pigaditou (3 (3) 0/3)
Athens, Attikon University General Hospital, ads, CIC 604, P. Tsirigotis (48 (49) 43/5)
Athens, Diagnostic & Therapeutic Center 'Hygeia', ads, CIC 643, G. Karianakis (5 (5) 0/5)
Athens, Evanghelismos Hospital, ads, CIC 622, D.Karakasis (51 (55) 38/13)
Athens, G. Gennimatas Hospital, ads, CIC 0, T.Marinakis, G. Gkortzolidis (9 (9) 0/9)
Athens, Hellenic Cancer Institute St. Savvas, ads, CIC 751, A.Pouli, J. Filis (41 (51) 0/41)
Athens, Laikon General Hospital, ads, CIC 438, J. Meletis, M. Angelopoulou (no report)
Heraklion, Crete, University Hospital, ads, H. Papadaki, C. Kalpadaki (no report)
Heraklion, Crete, University Hospital Heraklion, peds, CIC 10788, E. Stiakaki (2 (2) 0/2)
Patras, University Hospital of Patras, ads, peds, CIC 281, A. Spyridonidis, M. Liga (39 (39) 34/5)
Piraeus, Metaxa Cancer Hospital, ads, CIC 937, C. Kosmas (6 (11) 0/6)
Thessaloniki, The George Papanicolaou General Hospital, ads, CIC 561, I. Sakellari (97 (100) 55/42)

Hungary: (5 teams: 341 (359) 134/207)
Budapest, Central Hospital of Southern Pest, peds, CIC 824, G. Kriván (38 (41) 27/11)
Budapest, Dél-pesti Centrumkórház, National Institute of Hematology, ads, CIC 556, P.Remenyi, L. Gopcsa (176 (181) 83/93)
Debrecen, University of Debrecen, ads, CIC 648, A. Illes, L. Gergely (83 (89) 17/66)
Miskolc, GYEK, Child Health Centre, peds, CIC 599, R. Simon, A.Kelemen (11 (12) 7/4)
Pécs, University of Pécs, ads, CIC 682, A.Szomor (33 (36) 0/33)

Iceland: (1 team: 17 (18) 0/17)
Reykjavik, National University Hospital, ads, CIC 605, I. Hjálmarsdóttir, S. Reykdal (17 (18) 0/17)

Iran: (5 teams: 839 (862) 415/424)
Bandar Abbas, Hormozgan Medical Hospital, (ads), CIC 10185, M. Molavi (no report)
Shiraz, Shiraz University of Medical Sciences, Nemazee Hospital, ads, peds, CIC 188, M. Ramzi (171 (178) 55/116)
Tehran, Childrens Medical Centre, peds, CIC 856, Amir Ali Hamidieh (106 (109) 94/12)
Tehran, Mofid Children Hospital, peds, CIC 10190, B. Shahin Shamsian (41 (41) 35/6)
Tehran, Shariati Hospital, SCT Research Centre, ads, peds, CIC 633, A. Mousavi (318 (331) 163/155)
Tehran, Taleghani Hospital Blood and Marrow Transplantation Center, ads, CIC 916, M. Mehdizadeh, A. Hajifathali, M. Jahli (203 (203) 68/135)
Teheran, Mahak Children´s Cancer Hospital, peds, CIC 10224, A. Mehrvar (no report)
Urmia, Urmia Medical Sciences University, (ads), R. Asghari (no report)

Iraq: (1 team: 35 (35) 20/15)
Sulaimania Kurdistan, HIWA Cancer Hospital, ads, peds, CIC 847, D. Othman, D. Hassan (35 (35) 20/15)

Ireland: (4 teams: 256 (287) 91/165)
Dublin, Our Lady's Hospital of Sick Children, Crumlin, peds, CIC 774, A. O'Marcaigh (21 (27) 11/10)
Dublin, St. Vincent's Hospital, ads, CIC 541, K. Fadalla (25 (25) 0/25)
Dublin, St.James Hospital, ads, CIC 257, K.Flynn, P. Browne, P.J. Hayden (153 (171) 80/73)
Galway, Galway University Hospitals, ads, CIC 408, A. Hayat (57 (64) 0/57)

Israel: (10 teams: 752 (820) 388/364)
Beer Sheba, Soroka University Medical Center, ads, CIC 481, V. Stavi (25 (25) 0/25)
Haifa, Rambam Medical Center, ads, peds, CIC 345, T. Zuckerman (131 (151) 58/73)
Jerusalem, Hadassah University Hospital, ads, peds, CIC 258, P. Stepensky (81 (83) 46/35)
Petach-Tikva, Beilinson Hospital, ads, CIC 409, M. Yeshurun (89 (92) 45/44)
Petach-Tikva, Childrens Medical Center, peds, CIC 755, J. Stein (38 (52) 25/13)
Revohot, Kaplan Hospital, ads, CIC 327, L. Shvidel (8 (9) 0/8)
Tel Aviv, Dana-Dwek Children's Hospital, Sourasky Medical Centre, peds, CIC 670, R. Elhasid (22 (29) 16/6)
Tel Aviv, Tel Aviv Sourasky Medical Center, ads, CIC 161, R. Ram (101 (107) 45/56)
Tel Hashomer, Chaim Sheba Medical Cebter, peds, CIC 572, A. Toren (69 (75) 51/18)
Tel Hashomer, Sheba Medical Center, ads, CIC 754, A. Nagler, A. Shimoni (188 (197) 102/86)

Italy: (93 teams: 4,626 (5,326) 1,785/2,841)
Alessandria, S.S. Antonio e Biagio e C. Arrigo, ads, CIC 825, M. Ladetto, M. Corsetti, F. Salvi, S. Tamiazzo (53 (63) 26/27)
Ancona, Azienda Ospedale Riuniti di Ancona, ads, CIC 788, A. Olivieri (57 (60) 31/26)
Ancona, Azienda Ospedale Salesi Riuniti, peds, CIC 0, P. Pierani, I. Carloni (4 (4) 0/4)
Ascoli Piceno, Mazzoni Hospital, ads, CIC 119, P. Galieni (33 (41) 14/19)
Avellino, A.O.S. Guiseppe Moscati, ads, CIC 789, A. Risitano (34 (39) 14/20)
Aviano, CRO IRCCS Aviano, ads, CIC 162, M. Michieli, M. Rupolo, M. Mazzucato (25 (34) 0/25)
Bari, IRCCS Istituto Tumori "Giovanni Paolo II", ads, CIC 934, A. Guarini (25 (26) 0/25)
Bari, Universitá degli Studi di Bari, peds, CIC 649, G. Specchia, P. Carluccio (8 (8) 0/8)
Barletta, Hospital of Barletta, ads, CIC 555, G. Tarantini (21 (21) 0/21)
Bergamo, ASST Papa Giovanni XXIII, ads, CIC 658, A. Rambaldi (101 (106) 61/40)
Bologna, Instituto Ortopedico Rizzoli, peds, CIC 453, A. Paioli (4 (4) 0/4)
Bologna, Policlinico S. Orsola-Malpighi, peds, CIC 790, A. Prete (19 (20) 12/7)
Bologna, San Orsola-Malpighi Hospital, ads, CIC 240, F. Bonifazi (143 (155) 50/93)
Bolzano, Ospedale San Maurizio, ads, CIC 299, M. Casini, I. Cavattoni (77 (77) 27/50)
Brescia, Azienda Ospedaliera Spedali Civili Di Bresia, ads, peds, CIC 141, D. Russo (38 (41) 37/1)
Brescia, Azienda Spedali Civili, ads, peds, CIC 288, A. Re, A. Tucci (101 (130) 0/101)
Brescia, Ospedale dei Bambini Spedali Civili, peds, CIC 741, F. Porta, G. Gaia (17 (20) 13/4)
Brindisi, Perrino Hospital, ads, CIC 920, D. Pastore (48 (50) 23/25)
Busto Arsizio, Ospedale di Circolo di Busto Arsizio, ads, CIC 927, E. Todisco (7 (11) 0/7)
Cagliari, Binagh Hospital, Armando Businco Centre, ads, CIC 8111, G. La Nasa, E Vacca (53 (63) 23/30)
Cagliari, Ospedale per le Microcitemie, peds, CIC 8112, A. Piroddi (9 (11) 4/5)
Campobasso, Gemelli Molise, ads, CIC 1034, A. Bacigalupo (0 (0) 0/0)
Catania, Ospedale Ferrarotto, University of Catania, ads, peds, CIC 792, G. Milone, G. Moschetti, L. Lo Nigro (66 (78) 27/39)*
Civitanove Marche, Ospedale di Civitanova Marche, ads, CIC 419, R. Centurioni, M. Mirabile (10 (14) 0/10)
Como, Valduce Hospital, ads, CIC 473, M.Turrini, F. Alberio, V. Saccà (10 (22) 0/10)
Cremona, U.O. Ematologia CTMO, ads, CIC 0, A, Molteni, P. Spedini (4 (5) 0/4)
Cuneo, Azienda Ospedale "S. Croce e Carle", ads, CIC 606, N.Mordini (43 (47) 21/22)
Ferrara, University of Ferrara, ads, CIC 330, A. Cuneo (14 (22) 0/14)
Florence, Azienda Ospedaliera Universitaria di Careggi, ads, CIC 304, R. Saccardi, F. Bambi (104 (120) 46/58)
Florence, Azienda Ospedaliero Universitaria Meyer, peds, CIC 1003, V. Tintori (22 (24) 10/12)
Foggia, Azienda Ospedaliero Universitaria, ads, CIC 414, S. Capalbo, G. Spinosa (6 (7) 0/6)

Genova, Istituto Giannina Gaslini, peds, CIC 274, M. Faraci (28 (31) 17/11)
Genova, Ospedale San Martino, ads, CIC 217, E. Angelucci (121 (149) 52/69)
Latina, Ospedale Santa Maria Goretti, ads, CIC 379, E. Ortu la Barbera (31 (31) 0/31)
Lecce, Opsedale Vito Fazzi de Lecce, ads, CIC 868, N. Di Renzo (31 (37) 14/17)
Milan, Instituto Europeo di Oncologia, ads, CIC 331, R. Pastano. (42 (44) 19/23)
Milan, Ist. Nazionale Tumori di Milano, ads, peds, CIC 616, P. Corradini (94 (108) 15/79)
Milan, Istituto Clinico Humanitas IRCCS, ads, CIC 354, S. Bramanti, B. Sarina (106 (111) 47/59)
Milan, Istituto Scientifico H.S. Raffaele, ads, peds, CIC 813, F. Ciceri, M. Marcatti (122 (140) 68/54)
Milan, Ospedale di Niguarda, ads, CIC 294, G. Grillo, C. Vigano (78 (90) 36/42)*
Milan, University of Milan IRCCS, ads, CIC 265, G. Saporiti, S. Girelli (65 (78) 37/28)
Milan, Azienda Socio Sanitaria Territoriale Fatebenefratelli Saccco, (ads), C. Bianchi. M. Bruno Vente (no report)
Modena, University of Modena, ads, peds, CIC 543, F. Narni, P. Bresciani, G. Palazzi (41 (44) 19/22)
Monza, Ospedale San Gerardo, peds, CIC 279, A. Bondi, A. Rovelli (33 (34) 32/1)
Monza, Ospedale San Gerardo, Universita Di Milano-Bicocca, ads, CIC 544, P. Pioltelli, M. Parma (86 (86) 39/47)
Napels, AORN Cardarelli Hospital, ads, CIC 607, A. Picardi, M. Celentano, M. Pedata (56 (62) 20/36)
Napels, Frederico ll University, ads, CIC 766, F. Pane, G. Battipaglia (41 (41) 13/28)
Napels, Hospital Pausilipon, peds, CIC 341, V. Poggi, M. Ripaldi (21 (23) 10/11)
Napels, National Cancer Institute IRCCS, ads, CIC 839, G. Marcacci, A. Pinto (47 (62) 0/47)
Novara, Ospedale Maggiore della Carita, ads, CIC 867, G. Gaidano, L. Nassi (33 (48) 0/33)
Nuoro, Ospedale San Francesco, ads, CIC 793, A. Palmas, A. Uras (18 (19) 0/18)
Padova, Clinica di Oncoematologia Pediatrica, peds, CIC 285, E.Calore, A. Biff, C.Mainardi (31 (34) 21/10)
Padova, Istituto Oncologia Veneto IOV-IRCCS, ads, CIC 0, D.Marino (13 (15) 0/13)
Padova, Padua University Hospital, ads, CIC 853, L. Trentin (44 (57) 0/44)
Pagani, Hospital A. Tortora, ads, CIC 191, C. Califano (18 (18) 0/18)
Palermo, A.O.R. Villa Sofia Cervello, ads, CIC 392, R. Scimè (66 (79) 27/39)
Palermo, ARNAS Civico Di Cristina, ads, CIC 157, O. Ziino (10 (10) 0/10)
Palermo, Ospedale die Bambini, peds, CIC 109, O. Ziino (2 (2) 0/2)
Palermo, Ospedale 'La Maddalena', ads, peds, CIC 692, M. Musso, F. Porretto, A. Crescinanno (115 (144) 35/80)
Parma, University of Palma, ads, CIC 245, D. Vallisa, L. Prezioso (37 (41) 18/19)
Pavia, IRCCS Policlinico S. Matteo, ads, CIC 10061, P. Bernasconi, E.P. Alessandrino (73 (82) 32/41)
Pavia, IRCCS Policlinico San Matteo, ads, CIC 10062, P. Pedrazzoli (15 (44) 0/15)
Pavia, Policlinico IRCCS St. Matteo, peds, CIC 10063, M.Zecca, L. Kelly (25 (28) 24/1)
Perugia, Ospedale Santa Maria della Misericordia, ads, peds, CIC 794, A. Velardi, A. Carotti (98 (105) 35/63)
Pesaro, AORMN Hospital, ads, CIC 529, G.Visani (34 (43) 11/23)
Pescara, Ospedale Civile, ads, peds, CIC 248, S. Santarone, P. Di Bartolomeo (84 (84) 30/54)
Piacenza, Hospital Guglielmo da Saliceto, ads, CIC 163, D.Vallisa (25 (31) 14/11)
Pisa, University of Pisa, ads, peds, CIC 795, M. Petrini, G. Casazza, M.Pelosini (69 (72) 25/44)
Potenza, San Carlo Hospital, ads, CIC 861, M. Pizzuti, M. Cimminiello (23 (28) 3/20)
Ravenna, Romagna Metropolitan Transplant Network, ads, CIC 306, F. Lanza (103 (148) 0/103)
Reggio di Calabria, Grande Ospedale Metropolitano, Bianchi Melacrino Morelli, ads, peds, CIC 587, G. Messina, M. Martino (144 (179) 52/92)
Reggio Emilia, Arcispedale S. Maria Nuova, ads, CIC 660, F. Merli, L. Facchini (44 (51) 12/32)
Rionero in Vulture, IRCCS Referral Cancer Center of Basilicata, ads, CIC 185, Dr. Pietrantuono (11 (15) 0/11)
Rome, IRRCS Ospedale Bambino Gesu, peds, CIC 796, F. Locatelli, F. Galaverna (135 (172) 93/42)
Rome, Ospedale S. Camillo, ads, CIC 287, L. Rigacci (15 (15) 4/11)
Rome, Rome Transplant Network, ads, CIC 756, W. Arcese, P. De Fabritiis (167 (180) 54/113)
Rome, Università " La Sapienza", ads, peds, CIC 232, R. Foa, A.P. Lori, S. Capria (75 (75) 31/44)
Rome, Università Cattolica S. Cuore, ads, peds, CIC 307, S. Sica, P. Chiusolo, A. Bacigalupo (152 (181) 74/78)
Salerno, AOU San Giovanni di Dio e Ruggi D'Aragona Hospital, ads, CIC 928, C. Selleri, B. Serio (29 (30) 11/18)
San Giovanni Rotondo, Hospital Casa Sollievo Sofferenza, ads, CIC 526, AM. Carella (69 (82) 39/30)
Sassari, Universita Di Sassari, ads, CIC 870, F. Dore, L. Podda (10 (11) 0/10)
Siena, Azienda Ospedaliera Universitaria Senese, ads, CIC 321, G. Marotta, M. Tozzi (34 (39) 15/19)

Taranto, Institute of Haematologie, Ospedale Nord, ads, CIC 332, P. Mazza, G. Palazzo (31 (31) 8/23)
Torino, A.O.U Citta della Salute e della Scienza di Torino, ads, CIC 231, B. Bennedetto (101 (122) 29/72)
Torino, University Hospitals Torino, ads, peds, CIC 305, M.Berger,F. Fagioli,F. Carnevale,D,Cilloni,A.Cignetti (83 (90) 31/52)
Treviso, Presidio Ospedaliero Treviso, ads, CIC 415, F. Gherlinzoni (34 (52) 0/34)
Tricase (Lecce), Hospital C. Panico, ads, CIC 652, V. Pavone (38 (38) 15/23)
Trieste, Azienda Sanitaria Universitaria Integrata di Treste, ads, CIC 982, F. Zaja, G. Desabbata (11 (12) 0/11)
Trieste, Istituto per l'Infanzia, IRCCS Burlo Garofolo, peds, CIC 525, N. Maximova, M. Rabusin (11 (11) 10/1)
Udine, Azienda Ospedaliero Universitaria di Udine, ads, CIC 705, A. Sperotto, R. Fanin (109 (109) 73/36)
Varese, Ospedale di Circolo e Fondazione Macchi, ads, CIC 878, A. Ferrario. B. Bianchi (27 (33) 0/27)
Venice, Ospedale dell'Angelo, ads, CIC 502, C. Skert, M. Vespignani (41 (52) 14/27)
Verona, Policlinico G. B. Rossi, ads, peds, CIC 623, F. Benedetti, S. Cesaro (80 (80) 48/32)
Vicenza, Ospedale S. Bortolo, ads, CIC 797, C. Borghero, M. Ruggeri (40 (40) 20/20)

Jordan: (1 team: 217 (221) 113/104)
Amman, King Hussein Cancer Centre, ads, peds, CIC 580, A. Tbakhi (217 (221) 113/104)
Amman, Istishari Hospital, (ads), CIC 487, A. Ahmed Hussein (no report)

Kazakhstan: (1 team: 59 (59) 29/30)
Astana, National Research Center for Oncology and Transplantology, ads, CIC 0, V. Kemaikin (59 (59) 29/30)

Latvia: (1 team: 28 (28) 0/28)
Riga, Clinic Linezers, ads, CIC 0, S. Lejiniece, I. Trociukas (28 (28) 0/28)

Lebanon: (2 teams: 193 (202) 96/97)
Beirut, American University of Beirut, ads, peds, CIC 369, A. Bazarbachi (85 (94) 43/42)
Bsalim, Middle East University Hospital, ads, peds, CIC 477, A. Ibrahim (108 (108) 53/55)

Lithuania: (3 teams: 192 (224) 72/120)
Kaunas, University of Health Sciences Kauno Klinikos, ads, CIC 942, R. Gerbutavicius, D. Vaitiekus (47 (48) 16/31)
Vilnius, Santariskiu Klinikos, ads, CIC 644, L. Griskevicius, I. Trociukas (134 (163) 50/84)
Vilnius, University Childrens Hospital, peds, CIC 508, J. Rascon (11 (13) 6/5)

Luxembourg: (1 team: 24 (24) 0/24)
Luxembourg, Center Hospitalier, ads, CIC 0, S. De Wilde (24 (24) 0/24)

Macedonia: (1 team: 54 (54) 18/36)
Skopje, University Clinic for Haematology, ads, CIC 381, B. Georgievski (54 (54) 18/36)

The Netherlands: (14 teams: 1,497 (1,624) 608/889)
Amsterdam, Academic Med Centre, ads, CIC 247, J. Zsivos, E. Nur (85 (87) 48/37)
Amsterdam, Antoni Van Leeuwenhoek Hospital, ads, CIC 976, S. C. Linn (43 (46) 0/43)
Amsterdam, VU University Medical Center, ads, CIC 588, E. Meijer, G.J. Ossenkoppele (178 (197) 63/115)
Enschede, Medisch Spectrum Twente, ads, CIC 360, M.R. Schaafsma, MC. Legdeur (28 (29) 0/28)
Groningen, University Medical Centre UMCG, ads, CIC 546, M.R. De Groot, G. Choi (174 (174) 90/84)
Leiden, University Hospital, ads, peds, CIC 203, J.H. Veelken (155 (162) 122/33)
Maastricht, University Hospital, ads, CIC 565, G. Van Gorkom (133 (148) 41/92)
Nieuwegein, St. Antonius Hospital, ads, CIC 200, H.K. Koene, O. de Weerdt (43 (47) 0/43)
Nijmegen, University Hospital, ads, CIC 237, N. Schapp, T. De Witte (130 (142) 61/69)
Rotterdam, Erasmas MC Cancer Institute, ads, CIC 246, J.J. Cornelissen, (256 (281) 96/160)
The Hague, Haga Hospital Leyenburg, ads, CIC 547, M.R. Schipperus, S. Kersting (39 (47) 0/39)
Utrecht, Princess Maxima Centre for Ped Oncology, peds, CIC 352, M. Bierings (62 (77) 32/30)
Utrecht, University Medical Centre UMCU, ads, CIC 239, E. Petersen (137 (147) 55/82)

Zwolle, Isala Klinieken, ads, CIC 548, G.L. van Sluis (34 (40) 0/34)

Nigeria: (1 team: 7 (7) 5/2)
Benin, University Hospital Benin City, ads, CIC 0, N. Bazuaye (7 (7) 5/2)

Norway: (5 teams: 339 (420) 127/212)
Bergen, Haukelands Sjukhus, ads, CIC 197, A. Ahmed (41 (45) 3/38)
Oslo, Oslo University Hospital, ads, peds, CIC 235, T. Gedde-Dahl, J. Büchner (216 (272) 124/92)
Oslo, The Norwegian Radium Hospital, ads, CIC 782, J. Riise, M. Rodrigues, S. Kvaloy (40 (42) 0/40)
Tromso, University Hospital North Norway, ads, CIC 0, A. Vik, G. Knutsen (11 (21) 0/11)
Trondheim, St. Olavs Hospital, ads, CIC 0, O. Hjertner (31 (40) 0/31)

Poland: (16 teams: 1,279 (1,362) 588/691)
Bydgoszcz, Nicolaus Copernicus University, peds, CIC 764, J. Styczynski, R. Debski (28 (31) 21/7)
Cracow, Jagiellonian University CMUJ, ads, CIC 553, A. Skotnicki (83 (93) 38/45)
Cracow, University Children's Hospital JUMC, peds, CIC 507, J. Gozdzik (23 (25) 20/3)
Gdansk, Medical University, ads, peds, CIC 799, M. Bieniaszewska (150 (150) 77/73)
Gliwice, Maria Curie Memorial Cancer Centre, (ads), CIC 428, S. Giebel (no report)
Katowice, Silesian Medical Academy, ads, CIC 677, G. Helbig (271 (310) 130/141)
Lodz, Medical University of Lodz, ads, CIC 171, T. Robak (56 (56) 6/50)
Lublin, Childrens University Hospital, peds, CIC 678, K. Drabko, J. Kowalczyk (17 (19) 16/1)
Lublin, University Medical School, ads, CIC 695, T. Gromek, M. Wach, A. Walter-Croneck, W. Legiec (38 (40) 5/33)
Poznan, Poznan University of Medical Sciences, ads, CIC 730, L. Gil, A. Agnieszka (127 (127) 70/57)
Poznan, University of Medical Sciences, Pediatric Hematology, peds, CIC 641, J. Wachowiak (22 (22) 14/8)
Warsaw, Institue of Haematology and Blood Transfusion, ads, CIC 693, K.Halaburda, B.Nasilowska, A.Tomaszewska (93 (99) 51/42)
Warsaw, Maria Sklodowska Curie National Research Institute, ads, CIC 800, J. Walewski (81 (84) 0/81)
Warsaw, Military Institute of Health Services, ads, CIC 816, P. Rzepecki, K. Sulek (50 (50) 19/31)
Warsaw, Central Clinical Hospital, (ads), CIC 954, G. Basak, P. Rusicka, W. Wiktor-Jedrzejczak, P. Boguradzki (no report)
Wroclaw, Cape of Hope Medical University, peds, CIC 817, A. Chybicka, K. Kalwak, J. Owoc-Lempach (63 (69) 48/15)
Wroclaw, Lower Silesian Center, ads, CIC 538, A. Lange (38 (46) 17/21)
Wroclaw, University Hospital SPSK 1, ads, CIC 699, T. Wrobel (139 (141) 56/83)

Portugal: (6 teams: 499 (544) 153/346)
Coimbra, University Hospital, ads, CIC 905, C.Geraldes, A. Do Cén Teixeira, L. Ribeiro (57 (60) 0/57)
Lisbon, H. St. Antonio dos Capuchos, ads, CIC 826, A. Botelho de Sousa (55 (58) 0/55)
Lisbon, Hospital de Santa Maria, ads, CIC 636, F. Costa, F. Forjaz de Lacerda (64 (64) 33/31)
Lisbon, Instituto Portugues de Oncologia, ads, peds, CIC 300, M. Nuno (96 (107) 50/46)
Porto, Hospital St. Joao, ads, CIC 329, J. E. Guimaraes, F. Trigo (96 (103) 15/81)
Porto, Instituto Portugues de Oncologia, ads, peds, CIC 291, A. Campos (131 (152) 55/76)

Romania: (5 teams: 219 (231) 77/142)
Bucharest, Coltea Clinical Hospital, ads, CIC 912, A. Colita, A. Lupu, C. Ghimici (16 (17) 0/16)
Bucharest, Fundeni Clinical Centre, peds, CIC 935, A. Colita, L. Dumitrache (35 (36) 26/9)
Bucharest, Fundeni Clinical Institute, ads, CIC 427, A. Tanase (116 (124) 47/69)
Targu-Mures, Sectia Clinica de Hematologie si, ads, CIC 178, I. Benedek (32 (34) 1/31)
Timisoara, Emergency Hospital Louis Turcanu, ads, peds, CIC 174, M. Serban, C. Jinca (20 (20) 3/17)

Russia: (14 teams: 1,471 (1,605) 558/913)
Ekaterinburg, Regional Hospital No. 1, ads, CIC 0, T.S. Konstantinova, V.A. Shalaev (59 (59) 21/38)
Ekaterinburg, Royal Children's Hospital, peds, CIC 884, L. Fechina (33 (38) 26/7)
Moscow, Cancer Research Center N. N. Blokhin, ads, CIC 0, G. Petrova, K. Kirgizov (60 (64) 0/60)
Moscow, Central Clinical Hospital (CCHPA), ads, CIC 0, S. Shamansky (6 (7) 0/6)
Moscow, Federal Research Center for Pediatric Hematology, peds, CIC 694, A. Maschan, D. Balachov (153 (206) 138/15)
Moscow, FMBC Burnasyan, ads, CIC 0, A. Davtyan, A.E. Baranov (23 (29) 0/23)

Moscow, Main Military Clinical Hospital, ads, CIC 0, O.A. Rukavitsyn, V. Pop (6 (6) 0/6)
Moscow, N.N. Blokhin National Medical Research Center, peds, CIC 893, K. Kirgizov (118 (119) 22/96)
Moscow, National Pirogov Medical Centre, ads, CIC 0, V. Melnichenko, N. Mochkin (325 (325) 4/321)
Moscow, The Russian Children's Research Hospital, peds, CIC 411, E. Skorobogatova (71 (78) 58/13)
Moscow, Research Haematology Center of RAS, (ads), CIC 930, V.G. Savchenko (no report)
Novosibirsk, Institute of Clinical Immunolgy, ads, CIC 376, V. Sergeevuicheva (51 (51) 6/45)
Samara, Samara Kalinin Regional Hospital, ads, V.A. Rossiev (no report)
St. Petersburg, First State Pavlov Medical University, ads, peds, CIC 725, B.V. Afanasyev, L. Zubarovskaya (399 (446) 256/143)
St. Petersburg, Research Institute of Oncology na N.N. Petrov, ads, peds, CIC 845, S. Alekseev, I. Zyuzgin (114 (122) 22/92)
St. Petersburg, Russian Scientific and Research Institute of Haematology, ads, CIC 0, S. Voloshin (53 (55) 5/48)
St. Petersburg, Federal Centre V.A. Almazov, (ads), A. Zaritskey, D. Motorin (no report)

Saudi Arabia: (5 teams: 679 (734) 461/218)
Dammam, King Fahad Specialist Hospital, (ads, peds), CIC 441, H. Al-Hashmi (no report)
Jeddah, King Faisal Hospital, ads, peds, CIC 858, M. Bayoumi (83 (98) 50/33)
Riyadh, King Abdul Aziz Medical City, ads, peds, CIC 444, M. Al Zahrani (184 (199) 132/52)
Riyadh, King Fahad Medical City, ads, peds, CIC 159, M. Al-Harbi (76 (89) 39/37)
Riyadh, King Faisal Specialist Hospital, ads, peds, CIC 397, M. Al Jurf (207 (211) 118/89)
Riyadh, King Faisal Specialist Hospital, peds, CIC 981, M. Ayas, A. AlSeraihy (129 (137) 122/7)
Riyadh, Prince Sultan Military Medical City, (ads), CIC 818, S. Al Otaibi (no report)

Serbia: (4 teams: 169 (170) 49/120
Belgrade, Clinical Center of Serbia, ads, CIC 373, M. Todorovic Balint (98 (98) 20/78)
Belgrade, Military Medical Academy, ads, CIC 582, D. Stamatovic (42 (43) 16/26)
Belgrade, Mother and Child Health Institute, peds, CIC 358, D. Vujic (20 (20) 12/8)
Novi Sad, Clinical Center of Vojvodina, ads, CIC 655, A. Savic (9 (9) 1/8)

Slovakia: (5 teams: 192 (220) 87/105)
Banská Bystrica, Roosevelt Hospital, ads, CIC 333, I. Markuljak, E. Kralikova (15 (19) 0/15)
Bratislava, National Cancer Institute, ads, CIC 368, A. Vranovsky (64 (74) 15/49)
Bratislava, University Hospital, ads, CIC 610, M.Mistrik (66 (76) 53/13)
Bratislava, University Hospital, peds, CIC 684, J. Horàkovà, I. Bodova (28 (32) 19/9)
Kosice, University Hospital, ads, CIC 0, T. Guman, N. Stecova (19 (19) 0/19)

Slovenia: (1 team: 112 (136) 35/77)
Ljublijana, University Medical Centre, ads, peds, CIC 640, M. Sever, V. Rajic (112 (136) 35/77)

South Africa: (8 teams: 284 (297) 106/178)
Bloemfontein, Free State University Hospital, (ads), J. Malherbe (no report)
Cape Town, Constantiaberg Medical Clinic, ads, CIC 772, M. Du Toit (26 (26) 8/18)
Cape Town, Groote Schuur Hospital, ads, peds, CIC 512, E. Verburgh (33 (33) 14/19)
Cape Town, Netcare Kuils River Hospital, ads, peds, CIC 0, H. Koornhof (69 (71) 24/45)
Cape Town, Melomed Hospital Tokai, (ads, peds), S. Nahrwar (no report)
Durban, Inkosi Albert Luthuli Hospital, ads, peds, CIC 0, S. Parasnath (5 (5) 1/4)
Durban, Netcare Umhlanga Hospital, (ads), N. Sewpersad (no report)
Durban, Capital Haematology Hospital, (ads), J.P.Singh (no report)
Johannesburg, Chris Hani Baragwanath Hospital, ads, CIC 0, V. Philips, G. Stander (15 (15) 0/15)
Johannesburg, Wits Donald Gordon Medical centre, ads, peds, CIC 483, J. Thomson (28 (29) 14/14)
Pretoria, Netcare Pretoria East Hospital, ads, peds, CIC 456, D. Brittain, A. McDonald (87 (96) 37/50)
Randburg, Netcare Olivedale Hospital, ads, CIC 889, K. Gunther. D. Brittain (21 (22) 8/13)

Spain: 68 teams: 3,197 (3,438) 1,354/1,843)
Alicante, Hospital General, ads, CIC 0, P. Fernandez Albellan (34 (34) 0/34)
Alicante, Hospital Universitario Torrecardenas, ads, CIC 486, M.J. Garcia Perez (8 (9) 0/8)
Barakaldo Vizcaya, Hospital de Cruces, ads, peds, CIC 393, J. Garcia-Ruiz, J. Mateos-Mazon (55 (74) 0/55)
Barcelona, Hospital Clinic, ads, CIC 214, M. Rovira (105 (113) 51/54)

Barcelona, Hospital General Vall d'Hebron, ads, CIC 584, D. Valcarcel (57 (63) 35/22)
Barcelona, Hospital Germans Trias i Pujol, ads, CIC 613, J M. Ribera Santasusana (69 (70) 34/35)
Barcelona, Hospital M. Infantil, Vall d'Hebron, peds, CIC 422, C. Diaz de Heredia (37 (44) 29/8)
Barcelona, Hospital Mutua de Terrassa, ads, CIC 0, J. M. Marti Tutusaus (26 (34) 0/26)
Barcelona, Hospital Sant Joan de Deu, peds, CIC 668, I. Badell-Serra (20 (25) 18/2)
Barcelona, Institute Catala d'Oncologia, Hospital Duran i Reynals, ads, CIC 759, A. Mussetti,, A. Sureda (92 (95) 43/49)
Barcelona, Santa Creu i Sant Pau, ads, CIC 260, J. Sierra, S. Brunet, A. Esquirol (93 (97) 40/53)
Barcelona, Santa Creu i San Pau, peds, CIC 260, I. Badell Serra, M. Torrent (4 (4) 4/0)
Caceres, Hospital San Pedro de Alcantara, ads, CIC 0, J. Prieto, JM. Bergua (54 (54) 0/54)
Cadiz, University Hospital of Jerez de la Frontera, ads, CIC 0, R. Saldaña Moreno, S. Garzon-Lopez (55 (60) 30/25)
Castellon de La Plana, Hospital General de Castellon, ads, CIC 844, R. Garcia-Boyero (20 (21) 0/20)
Cordoba, Hospital Reina Sofia, ads, peds, CIC 238, C. Herrere Arroyo, C. Martin Calvo, V. Martin Palanco (64 (64) 28/36)
Galdakao, Hospital de Galdakao, ads, CIC 0, J.Ojanguren, T.Carrascosa, K.Atutxa (23 (23) 0/23)
Girona, Institut Catala d'Oncologia, Josep Trueta, ads, CIC 433, D. Gallardo (14 (14) 0/14)
Granada, Hospital Virgen de la Nieves, ads, CIC 559, M. Jurado Chacon (74 (78) 32/42)
Jaen, Hospital Cuidad de Jaen, ads, CIC 0, F. Almagro Torres (21 (21) 0/21)
La Coruna, Complejo Hospitalario de A Coruna, ads, CIC 361, J P. Torres Carrete, M R. Varela Gomez (53 (59) 20/33)
La Laguna, Tenerife, University Hospital Canary Isles, (ads), M.T. Hernandez-Garcia, B. Soris S. Barbara (no report)
Las Palmas Canary Isles, Hospital de Gran Canaria 'Dr. Negrin', ads, CIC 537, M.del Mar Perera, A.Suarez, H.Luzardo (72 (79) 41/31)*
Las Palmas Canary Isles, Hospital Insular, ads, CIC 0, J. Gonzalez-San Miguel (23 (23) 0/23)
Leon, Hospital Universitario de Leon, ads, CIC 426, N de Las Heras (16 (16) 0/16)
Lleida, Hospital Arnau de Vilanova, ads, CIC 885, A. Garcia Guinon (16 (16) 0/16)
Logrono, Hospital San Pedro, La Rioja, ads, CIC 917, M. Najera Irazu, M. Hermosilla (15 (15) 0/15)
Lugo, Hospital Lucus Augusti, ads, CIC 0, J. Arias Sampedro (11 (12) 0/11)
Madrid, Fundacion Jimenez Diaz, ads, CIC 309, JL. Lopez-Lorenzo (57 (60) 20/37)
Madrid, Hospital de la Princesa, ads, CIC 236, A. Figuera, A. Alegre (45 (46) 27/18)
Madrid, Hospital Doce de Octubre, ads, peds, CIC 382, J. Martinez, M.L.Paciello (89 (89) 28/61)
Madrid, Hospital General Universitario Gregorio Maranon, ads, CIC 819, J.L. Diez-Martin, P. Balsalobre (63 (71) 49/14)
Madrid, Hospital Niño Jesus, peds, CIC 732, M.A. Diaz (48 (63) 38/10)
Madrid, Hospital Principe Asturias, Alcala de Henares, ads, CIC 0, Dr. Lopez Rubio, E. Magro Mazo (15 (15) 0/15)
Madrid, Hospital Ramon y Cajal, ads, CIC 615, J. Lopez-Jimémez (98 (98) 46/52)
Madrid, Hospital Severo Ochoa, Leganés, ads, CIC 0, P. Sanchez Godoy (6 (6) 0/6)
Madrid, Hospital Univeristario Materno Infantil Gregorio Maranon, peds, CIC 410, C. Belendez (13 (14) 11/2)
Madrid, Hospital Universitario La Paz, ads, peds, CIC 734, A. Perez-Martinez, R. De Paz (46 (55) 34/12)
Madrid, Hospital Universitario Puerta de Hierro, ads, CIC 728, JR Cabrera Martin, G. Bravo (48 (51) 22/26)
Madrid, Hospital Universitario Quironsalud and Hospital Moncloa, ads, CIC 0, JM. Fernadez-Ranada, A. Escudero, M. Chamarro (40 (40) 5/35)
Madrid, Hospital Universitario San Carlos, ads, CIC 0, M.Paz Martin, C. Benavente (9 (9) 0/9)
Madrid, Hospital Universitario Sanitas La Zarzuela, ads, CIC 779, R. De la Camara (6 (6) 0/6)
Madrid, Hospital Universitario de Getafe, (ads), L.Garcia Alonson, F.Oña Compan, N.Somolinos, C.Monteserin (no report)
Madrid, Hospital Universitario Sanchinarro, (ads), J. Pérez de Oteyza (no report)
Malaga, Hospital Regional Malaga, ads, peds, CIC 576, M-J. Pascual-Cason (126 (137) 72/54)
Malaga, Hospital Virgen de la Victoria, ads, CIC 476, A. Rosell Mas (18 (18) 0/18)
Murcia, Hospital General Universitario Morales Meseguer, ads, peds, CIC 735, I. Heras, V. Vicente-Garcia (50 (57) 17/33)
Murcia, Hospital Virgen de la Arrixaca, ads, peds, CIC 323, JM. Moraleda, A. Sanchez-Salinas (56 (61) 21/35)
Orense, Com. Hospital Cristal-Pinor, (ads), J-L. Sastre-Moral (no report)
Oviedo, Hospital Covadonga, Central Asturias, ads, peds, CIC 642, S. Gonzalez-Müniz (74 (77) 34/40)

Palma de Mallorca, Hospital son Llatzer, ads, CIC 110, J. Bargay-Lleonart (20 (22) 0/20)
Palma de Mallorca, Hospital Uni. Son Espases, ads, peds, CIC 722, A. Sampol (52 (55) 20/32)
Pamplona, Clinica Universitaria de Navarra, ads, peds, CIC 737, J. Rifon (56 (58) 27/29)
Pamplona, Hospital de Navarra, ads, CIC 577, T. Zudaire (40 (44) 16/24)
Pontevedra, Hospital Montecelo, ads, CIC 0, A-M. Dios Loureiro (14 (15) 0/14)
Salamanca, Hospital Clinico, ads, peds, CIC 727, D. Caballero (141 (154) 81/60)
San Sebastian, Hospital Universitario Donostia, ads, peds, CIC 0, C. Vallejo Llamas,JJ Ferreiro Martinez (72 (82) 42/30)
Santander, Hospital Universitario Marqués de Valdecilla, ads, peds, CIC 242, M. Colorado Araujo (68 (78) 42/26)
Santiago de Compostela, Hospital Clinico Universitario, ads, peds, CIC 570, J.L. Bello Lopez (61 (70) 33/28)
Sevilla, Hospital Universitario Virgen del Rocio, ads, peds, CIC 769, J. A. Pérez-Simón (171 (172) 80/91)
Tarragona, Hospital Joan XXIII de Tarragona, ads, CIC 0, C. Talarn Forcadell (27 (27) 0/27)
Tenerife Canary Isles, Hospital N. S. De la Candelaria, ads, CIC 0, J. Garcia-Talavera, J. Breña, P. Rios Rull (29 (33) 0/29)
Valencia, Hospital Arnau de Vilanova de Valencia, ads, CIC 0, A. Lopez Martinez (14 (14) 0/14)
Valencia, Hospital Clinico de Valencia, ads, peds, CIC 282, C. Solano (79 (85) 38/41)
Valencia, Hospital Doctor Peset, ads, CIC 0, P. Ribas-Garcia, A. Garcia Feria (15 (18) 0/15)
Valencia, Hospital Universitario La Fe, ads, CIC 663, J. Sanz, G.F.Sanz (140 (149) 97/43)
Valencia, Hospital Universitario La Fe, peds, CIC 653, J.M. Fernandez Navarro (18 (24) 11/7)
Valencia, Instituto Valenciano de Oncologia, ads, CIC 0, A. Avaria, C. Salazar (6 (6) 0/6)
Valladolid, Hospital Rio Hortega, ads, CIC 611, J. Garcia Frade (27 (28) 8/19)
Vigo, CHUVI Hospital Alvaro Cunqueiro, ads, CIC 421, C. Albo Lopez (47 (52) 21/26)
Zaragoza, Clinico Universitario Lozano Blesa, ads, CIC 0, L. Palomera Bernal (21 (21) 0/21)
Zaragoza, Hospital Miguel Servet, ads, peds, CIC 0, P. Delgado (41 (41) 9/32)

Sweden: (7 teams: 713 (812) 289/424)
Goteborg, CHECT Sahlgrenska University Hospital, ads, peds, CIC 289, J. Johansson, K. Mellgren (121 (144) 46/75)
Linköping, University Hospital, ads, CIC 740, A. Bergendahl Sandstedt (82 (83) 22/60)
Lund, University Hospital, ads, peds, CIC 283, S. Lenhoff, J. Toporski (126 (141) 64/62)
Örebro, Medical Center Hospital, ads, CIC 738, P. Kozlowski (19 (33) 0/19)
Stockholm, Karolinska University Hospital, ads, peds, CIC 212, S. Mielke, J. Winiarski, P. Ljungman (186 (188) 91/95)
Umea, Umea University Hospital, ads, CIC 731, C. Isaksson (54 (65) 15/39)
Uppsala, University Hospital, ads, peds, CIC 266, K. Carlson, N. Jackmann (125 (158) 51/74)

Switzerland: (10 teams: 679 (778) 262/417)
Aarau, Kantonsspital Aarau, ads, CIC 316, S. Gerull (33 (38) 0/33)
Basel, Universitätsspital Basel, ads, peds, CIC 202, J. Passweg, D. Heim, J. Halter (143 (152) 116/27)
Bellinzona, Ospedale San Giovanni, ads, CIC 829, L. Wannesson (17 (23) 0/17)
Bern, Inselspital, ads, peds, CIC 221, T. Pabst, J. Rössler, G. Baerlocher (121 (147) 0/121)
Geneva, Hôpital Cantonal Universitaire, ads, peds, CIC 261, Y. Chalandon, M. Ansari (63 (68) 63/0)
Lausanne, CHUV, ads, CIC 820, M. Duchosal (81 (87) 0/81)
St. Gallen, Kantonsspital, ads, CIC 324, F. Hitz (41 (46) 0/41)
Zurich, Hospital Hirslanden, ads, CIC 638, Ch. Renner (24 (29) 0/24)
Zurich, Universitäts Kinderklinik, peds, CIC 334, T. Güngör (23 (29) 23/0)
Zurich, University Hospital, ads, peds, CIC 208, U. Schanz, G. Nair, A. Müller (133 (159) 60/73)

Syria: (1 team: 14 (14) 7/7)
Damascus, Tishreen Hospital, ads, peds, CIC 10853, De. S. Elias Soulaiman (14 (14) 7/7)

Tunisia: (1 team: 103 (105) 49/54)
Tunis, National BMT Centre, ads, peds, CIC 183, B. Othman Tarck (103 (105) 49/54)

Turkey: (73 teams: 4,448 (4,642) 2,258/2,190)
Adana, Adana Acibadem Hospital, peds, CIC 454, A. Antmen (60 (60) 60/0)
Adana, Balcali Hospital, Cukurova University, ads, CIC 462, B. Güvenc (23 (24) 14/9)
Adana, Baskent University Adana, ads, CIC 589, H. Ozdogu, C. Boga, S. Asma (101 (103) 55/46)

Adana, Cukurova University Balcali Hospital, peds, CIC 0, G. Inan (48 (48) 43/5)
Ankara, Ankara Baskent Hospital, ads, CIC 0, S. Zeynep Aki, E. Koca (16 (17) 4/12)
Ankara, Ankara Bayindir Hospital, ads, CIC 412, A.Ural (32 (36) 18/14)
Ankara, Ankara University Faculty of Medicine, Dikimevi, ads, CIC 617, P. Topguoglu, G. Gürmann, M. Kurt Yüksel (80 (93) 33/47)
Ankara, Dr. A. Yurtaslan Oncology Training and Research Hospital, ads, CIC 0, F. Altuntas, M. Sinan Dal (104 (104) 51/53)
Ankara, Gazi University Medical School, Besevler, ads, CIC 169, Z.N. Ozkurt, Ö. Karacaoglu (51 (62) 26/25)
Ankara, Gazi University Medical School, Besevler, peds, CIC 182, U. Kocak (13 (13) 11/2)
Ankara, Hacettepe Ihsan Dogramaci Childrens Hospital, peds, CIC 399, D. Uckan-Cetinkaya, B. Kuskonmaz (19 (19) 19/0)
Ankara, Hacettepe University Medical School, Sihhiye, ads, CIC 168, H. Goker (59 (59) 30/29)
Ankara, Liv Hospital, ads, CIC 0, O. Nevruz (90 (90) 38/52)
Ankara, Lösante Hospital, ads, peds, CIC 0, A.Emin Kurekci (38 (39) 34/4)
Ankara, Memorial Hospital Ankara, ads, CIC 0, F. Avcu (47 (48) 25/22)
Ankara, Numune Education and Research Hospital, ads, CIC 691, G. Özet (50 (50) 14/36)
Ankara, Ozel Koru Hospital, ads, CIC 0, A. Ugur Bilgin (37 (37) 18/19)
Ankara, Private Medicana International Hospital, ads, CIC 0, U. Sahin (119 (130) 58/61)
Ankara, University of Ankara, Cebeci, peds, CIC 620, T. Lleri, E. Unal (18 (19) 17/1)
Ankara, Yildirim Bayazit Training and Research Hospital, ads, CIC 0, M. Albayrak (9 (9) 0/9)
Ankara, SBU Gülhane Training and Research Hospital, (ads), N. Karadurmus, O. Gürsel, R. Acar (no report)
Ankara, Children's State Hospital, (peds), CIC 436, B. Tunc (no report)
Antalya, Akdeniz University Medical School, peds, CIC 618, A. Kupesi (51 (56) 46/5)
Antalya, Akdeniz University School of Medicine, ads, CIC 685, L. Undar (49 (52) 21/28)
Antalya, Medical Park Antalya Hospital, Lara, peds, CIC 911, A. Yesilipek (93 (111) 90/3)
Antalya, Medstar Antalya Hospital, Cakirlar, ads, CIC 864, I. Karadogan (105 (106) 30/75)
Atakum, Özel Sansung Medicalpark Hospital, peds, CIC 881, H.E. Ozyurek, E. Sahin (0 (0) 0/0)
Antalya, Antalya Education and Research Hospital, (ads), CIC 914, I. Nizam Özen (no report)
Aydin, Adnan Menderes University Medical Faculty, ads, CIC 187, Z. Bolaman, I. Yavasoglu (48 (51) 14/34)
Bursa, Uludag University School of Medicine, ads, CIC 0, V. Özkocaman (45 (45) 13/32)
Bursa, Uludag University School of Medicine, peds, CIC 0, AM. Günes, M. Evim (14 (14) 13/1)
Denizli, Pamukkale University Hospital, ads, CIC 0, S. Kabukcu (44 (44) 0/44)
Diyarbakir, Dicle University Faculty, ads, CIC 0, O. Ayyildiz (17 (17) 0/17)
Erzurum, Atatürk University, ads, CIC 0, G. Sincan (12 (16) 4/8)
Eskisehir, Osmangazi University, ads, CIC 0, E. Gündüz (35 (44) 7/28)
Istanbul, Acibadem University Altunizade Hospital, ads, CIC 468, S. Ratip, E. Ovali (87 (87) 37/50)
Istanbul, Acibadem University Altunizade Hospital, peds, CIC 457, G. Öztürk (32 (32) 31/1)
Istanbul, Acidadem Atakent Hospital, ads, CIC 0, S. Sami Karti, A. Uzay (189 (189) 74/115)
Istanbul, Bahcelievler Medical Park Hospital, ads, CIC 0, G. Sucak (91 (103) 42/49)
Istanbul, Bahcelievler Memorial Hospital HSCTU Center, ads, CIC 0, E. Tekgündüz (85 (92) 53/32)
Istanbul, Cerrahpasa Medical Faculty, ads, CIC 761, T. Soysal, M. Cem Ar, T. Elverdi (65 (72) 21/44)
Istanbul, Emsey Hospital, ads, CIC 355, S. Omay, Y. Ünsal (65 (65) 35/30)
Istanbul, Florence Nightingale Sisli Hospital, ads, CIC 994, M. Arat (90 (90) 41/49)
Istanbul, Hisar Intercontinental Hospital, ads, CIC 0, M. Köroğlu, E. Büyük (61 (61) 32/29)
Istanbul, Istanbul Medipol University, ads, CIC 445, L. Gül Kaynar (103 (103) 37/66)
Istanbul, Istanbul Medipol University, peds, CIC 446, Y. Yaman, S. Anak (41 (41) 32/9)
Istanbul, Koç University Hospital, ads, CIC 943, O. Akay (87 (89) 27/60)
Istanbul, Marmara University Hospital, ads, CIC 714, T. Firatli-Tuglular, T. Toptas (28 (28) 10/18)
Istanbul, Medical Park Bahcelievler Hospital, peds, CIC 4482, T. Fisgin, C. Bozkurt (78 (92) 64/14)
Istanbul, Medical Park Goztepe, peds, CIC 0, G. Karasu, SC. Kilic (102 (111) 97/5)
Istanbul, Medical Park Göztepe, ads, CIC 0, C. Adigüzel (133 (133) 48/85)
Istanbul, Medicana International Hospital, ads, CIC 919, Y. Koc (51 (54) 26/25)
Istanbul, Sisli Memorial Hospital, ads, CIC 0, S. Izmir Güner (102 (102) 44/58)
Istanbul, Sisli Memorial Hospital, peds, CIC 0, A. Tanyeli (19 (20) 16/3)
Istanbul, University of Istanbul, ads, CIC 760, I. Yonal-Hindilerden, M. Aktan (43 (47) 14/29)
Istanbul, Yeditepe University Hospital, ads, CIC 416, A. Özkan (39 (41) 15/24)
Istanbul, Yeditepe Universtity Hospital, peds, CIC 0, N. Yalman, C. Timur (8 (8) 7/1)

Istanbul, Yeniyuzyil University, Gaziosmanpasa Hospital, ads, CIC 475, H. Goksoy (125 (125) 52/73)
Istanbul, Yeniyuzyil University, Gaziosmanpasa Hospital, peds, CIC 459, B. Malbora (68 (68) 64/4)
Istanbul, Kolan International Hospital, ads, E. Kurtoglu (no report)
Istanbul, Medical Park Hospitals, (ads), CIC 919, B. Eser (no report)
Izmir, Dokuz Eylul University, ads, peds, CIC 688, G. H. Özsan. H. Ören (39 (39) 15/24)
Izmir, Ege University Medical Faculty, Bornova, ads, CIC 628, F. Vural, G. Saydam, N. Soyer (47 (47) 22/25)
Izmir, Ege University Medical Faculty, Bornova, peds, CIC 621, S. Kansoy (38 (42) 29/9)
Izmir, Izmir Bozyaka Hospital, ads, CIC 0, O. Bilgir, D. Cekdemir (28 (28) 0/28)
Izmir, Medicalpark Private Hospital, Karsiyaka, ads, peds, CIC 0, S.Cagirgan, Kahraman, C. Acarlar (97 (102) 59/38)
Izmir, Tepecik Research and Educational Hospital, peds, CIC 0, H. Öniz (4 (4) 4/0)
Kayseri, Erciyes University Faculty of Medicine, peds, CIC 913, M. Karakukcu (50 (56) 36/14)
Kayseri, Erciyes University Hospital, ads, CIC 627, A. Unal, M. Cetin (91 (95) 36/55)
Kocaeli, Anadolu Medical Center Hospital, ads, CIC 440, Z. Gülbas (311 (335) 185/126)
Kocaeli, Kocaeli University Hospital, ads, CIC 0, P. Tarkun (28 (28) 0/28)
Kocaeli, Kocaeli University Hospital, peds, CIC 0, E. Zengin (7 (7) 5/2)
Konya, Necmettin Erbakan, Meram University Medical Hospital, ads, CIC 0, Ö. Ceneli (24 (25) 0/24)
Malatya, Inönu University Hospital, peds, CIC 0, A. Akyay (20 (20) 18/2)
Malatya, Inonu University Targut Özal Medical Centre, ads, CIC 0, M. Ali Erkurt, E. Kaya (277 (277) 97/180)
Manisa, Celal Bayer University, ads, CIC 0, I. Aydogdu (11 (11) 1/10)
Samsun, Ondokuz Mayis University, peds, CIC 0, C. Albayrak (16 (16) 12/4)
Trabzon, KTU Farabi Hospital, ads, CIC 0, E. Nas, M. Sonmez (41 (41) 14/27)

Ukraine: (5 teams: 204 (222) 48/156)
Cherkasy, Cherkasy Regional Hospital, ads, peds, CIC 0, V. Paramonov (35 (35) 4/31)
Kiev, Kiev BMT Center MNPE KBMTC, ads, peds, CIC 0, V. Khomenko (68 (82) 2/66)
Kiev, National Cancer Centre, ads, CIC 10832, K. Filonenko, E. Kushcheviy (30 (30) 0/30)
Kiev, National Cancer Institute, peds, CIC 0, S. Pavlyk (18 (21) 0/18)
Kiev, National Pediatric Specialized Hospital, OHMATDYT, peds, CIC 10109, O. Lysytsia (53 (54) 42/11)

United Kingdom: (48 teams: 3,862 (4,189) 1,493/2,369)
Aberdeen, The Royal Infirmary, ads, CIC 344, D.J.Culligan (29 (31) 0/29)
Bath, Royal United Hospital, ads, CIC 619, J. Crowe (22 (22) 0/22)
Belfast, Belfast City Hospital Trust, ads, CIC 268, D. Finnegan (68 (71) 14/54)
Birmingham, Queen Elizabeth Hospital, ads, peds, CIC 387, R. Malladi, P. Shankara (260 (261) 82/178)
Birmingham, The Birmingham Children's Hospital, peds, CIC 781, S. Lawson (32 (34) 21/11)
Blackpool, Victoria Hospital, ads, CIC 832, M.P. Macheta (42 (46) 0/42)
Bournemouth, Royal Bournemouth Hospital, ads, CIC 765, R. Hall (24 (24) 0/24)
Bristol, Avon and Royal Hospital for Sick Children, ads, peds, CIC 386, D. Marks, S. Robinson (112 (118) 75/37)
Cambridge, Addenbrooke's Hospital, ads, CIC 566, C.Crawley,J Craig (132 (151) 64/68)
Cardiff/Swansea, University Hospital of Wales, ads, peds, CIC 303, K.M.O. Wilson, P. Connor, W. Ingram (104 (121) 36/68)
Cheltenham, Cheltenham General Hospital, ads, CIC 398, A. Rye (13 (13) 0/13)
Coventry, University Hospital Coventry & Warwickshire NHS Trust, ads, CIC 322, F. Jones (25 (27) 0/25)
Dudley, Dudley NHS Trust, ads, CIC 405, S. Fernandes (4 (5) 0/4)
Dundee, Ninewells Hospital, ads, CIC 719, D. Meiklejohn (17 (17) 0/17)
Edinburgh, The Western General Hospital, ads, CIC 228, A. J.M. Broom, F.Scott, P.Roddie (52 (61) 0/52)
Exeter, Royal Devon and Exeter Hospital, ads, CIC 571, P. Kerr (25 (25) 0/25)
Glasgow, Beatson, West of Scotland Cancer Centre, ads, peds, CIC 244, I.G. McQuaker, A. Parker, A. Barrett (149 (153) 78/71)
Glasgow, Royal Hospital for Children, peds, CIC 707, B. Gibson (14 (14) 10/4)
Leeds, Yorkshire Hospitals NHS Trust, ads, peds, CIC 254, M. Gilleece, J.Ashcroft. R. Patmore (197 (217) 77/120)
Leicester, Royal Infirmary Hospital, ads, CIC 713, M. Martin (70 (74) 21/49)
Liverpool, Alder Hay, peds, CIC 773, M. Caswell (1 (1) 0/1)
Liverpool, Royal Liverpool University Hospital, ads, CIC 501, A. Patel, R. Salim (69 (74) 28/41)

London, Great Ormond Street Hospital, peds, CIC 243, P. Veys (85 (90) 61/24)
London, Hammersmith Hospitals NHS Trust, ads, CIC 205, J.Apperley, E. Kanfer, D. Slade, R. Szydlo (122 (137) 58/64)
London, King's College Hospital, ads, CIC 763, G. Mufti, V. Potter (170 (177) 74/96)
London, London Bridge Hospital, ads, CIC 460, M. Kazmi (18 (18) 0/18)
London, Royal Marsden Hospital, ads, peds, CIC 218, M. Potter (221 (253) 89/132)
London, St Mary's Hospital, peds, CIC 866, J de La Fuente (23 (23) 23/0)
London, St. Bartholomew's and the Royal London NHS Trust, ads, CIC 768, J. Gribben, S. Montoto,J. Cavenagh, S. Agrawal (105 (118) 33/72)
London, St. George's Hospital, ads, CIC 539, M. Koh, M. Klammer (50 (55) 16/34)
London, The London Clinic, ads, CIC 263, M. Potter (34 (34) 7/27)
London, University College Hospital, ads, peds, CIC 224, B. Carpenter (313 (346) 102/211)
Manchester, Central Manchester NHS Trust, peds, CIC 521, R. F. Wynn (56 (73) 47/9)
Manchester, Christie NHS Trust Hospital, ads, CIC 780, A.Bloor (116 (118) 51/65)
Manchester, The Royal Infirmary, ads, CIC 601, E. Tholouli (142 (142) 75/67)
Newcastle upon Tyne, Freeman Hospital, ads, peds, CIC 276, M. Collins, M. Slatter (210 (218) 98/112)
Norwich, The Norfolk and Norwich University Hospital, ads, CIC 391, M. Lawes (30 (31) 0/30)
Nottingham, Nottingham City Hospital, ads, peds, CIC 717, J.L. Byrne (154 (166) 58/96)
Oxford, Cancer and Haematology Centre, Churchill Hospital, ads, CIC 255, A. Peniket,T.Littlewood,A.Brown,G.Collins (126 (126) 60/66)
Oxford, John Radcliffe Children's Hospital, peds, CIC 603, G. Hall (6 (10) 0/6)
Plymouth, University Hospitals Plymouth NHS Trust, ads, CIC 823, H.M.Hunter (78 (82) 30/48)
Poole, Poole Hospital NHS Foundation Trust, ads, CIC 458, J. Fergus (9 (10) 0/9)
Sheffield, Childrens NHS Foundation, peds, CIC 933, K. Patrick (25 (35) 15/10)
Sheffield, Teaching Hospitals NHS Trust , Children's Hospital, ads, CIC 778, J.Snowden (114 (133) 42/72)
Southampton, University Hospital Southampton NHS Foundation Trust, ads, peds, CIC 704, K. Orchard, D. Richardson (157 (195) 48/109)
Stoke-on-Trent, University Hospitals of North Midlands, ads, CIC 394, S. Pillai, R. Chasty (19 (21) 0/19)
Swindon, Great Western Hospital, ads, CIC 608, N. E. Blesing (8 (8) 0/8)
Taunton, Taunton and Somerset NHS Foundation Trust, ads, CIC 708, S. Bolam (10 (10) 0/10)

Europe total: 694 centres: (43,109 (47,412) 18,589/24,520)
*EBMT MEDAB data extract
